# Bevacizumab Treatment for Low-Grade Serous Ovarian Cancer: A Systematic Review

**DOI:** 10.3390/curroncol30090592

**Published:** 2023-09-03

**Authors:** Caitlin Lazurko, Revital Linder, Kate Pulman, Genevieve Lennox, Tomer Feigenberg, Rouhi Fazelzad, Taymaa May, Tiffany Zigras

**Affiliations:** 1 Temerty Faculty of Medicine, University of Toronto, Toronto, ON M5S 1A8, Canada; 2 Department of Obstetrics and Gynecology, Rambam Health Care Campus, Haifa 3109601, Israel; 3 Division of Gynecologic Oncology, Trillium Health Partners, Credit Valley Hospital, Mississauga, ON L5M 2N1, Canada; 4 Department of Library and Information Services, University Health Network Library and Information Services, Princess Margaret Cancer Centre, Toronto, ON M5G 2C4, Canada; 5 Division of Gynecologic Oncology, Department of Surgical Oncology, Princess Margaret Cancer Center, University Health Network, Toronto, ON M5G 2M9, Canada

**Keywords:** low-grade serous ovarian cancer, bevacizumab, overall response rate

## Abstract

Serous epithelial ovarian cancer, classified as either high-grade (90%) or low-grade (10%), varies in molecular, histological, and clinicopathological presentation. Low-grade serous ovarian cancer (LGSOC) is a rare histologic subtype that lacks disease-specific evidence-based treatment regimens. However, LGSOC is relatively chemo-resistant and has a poor response to traditional treatments. Alternative treatments, including biologic therapies such as bevacizumab, have shown some activity in LGSOC. Thus, the objective of this systematic review is to determine the effect and safety of bevacizumab in the treatment of LGSOC. Following PRISMA guidelines, Medline ALL, Cochrane Central Register of Controlled Trials, Cochrane Database of Systematic Reviews, Embase all from the OvidSP platform, ClinicalTrials.gov, International Clinical Trials Registry Platform, International Standard Randomised Controlled Trial Number Registry were searched from inception to February 2022. Articles describing bevacizumab use in patients with LGSOC were included. Article screening, data extraction, and critical appraisal of included studies were completed by two independent reviewers. The effect of bevacizumab on the overall response rate, progression-free survival, overall survival, and adverse effects were summarized. The literature search identified 3064 articles, 6 of which were included in this study. A total of 153 patients were analyzed; the majority had stage IIIC cancer (56.2%). The overall median response rate reported in the studies was 47.5%. Overall, bevacizumab is a promising treatment for LGSOC, with response rates higher than traditional treatment modalities such as conventional chemotherapy, and is often overlooked as a treatment tool. A prospective clinical trial evaluating the use of bevacizumab in LGSOC is necessary to provide greater evidence and support these findings.

## 1. Introduction

Low-grade serous ovarian cancer (LGSOC) is a rare form of cancer, accounting for about 10% of all epithelial ovarian cancers [1,2,3]. It differs from high-grade serous ovarian cancer histologically, molecularly, as well as in its clinical presentation. LGSOCs can develop de novo or from borderline ovarian tumors, whereas high-grade serous ovarian cancers are thought to be associated with tubal intraepithelial carcinoma [4,5]. LGSOCs often present with mutations in the KRAS, BRAF, or ERBB2 genes but typically will have an intact TP53 gene. This differs from high-grade serous ovarian cancers, which typically have a TP53 mutation. LGSOCs also have lower levels of chromosomal instability compared to high-grade serous ovarian cancers and a slower rate of tumor development [4]. Compared to high-grade serous ovarian cancer, patients with LGSOC present at a younger age have a more indolent course and higher rates of chemoresistance [2,3,4,6,7,8]. Moreover, many treatment modalities that have been successful in treating high-grade serous ovarian cancer have not been effective in the LGSOC population. Currently, the National Comprehensive Cancer Network (NCCN) suggests treating patients with advanced-stage LGSOC with cytoreductive surgery, followed by chemotherapy and/or hormone therapy [9]. However, the response rate to chemotherapy remains low, around 4% [7,8,10]. Thus, LGSOC-specific treatments are necessary to manage this rare form of cancer. Fortunately, LGSOC treatments are rapidly evolving as new therapies targeting the distinct features of LGSOC have been identified.

While LGSOC is a relatively indolent cancer, recurrence occurs in about 80% of patients [11]. Patients with advanced disease at presentation and at the time of recurrence are commonly treated with a combination of surgical cytoreduction followed by adjuvant therapy such as chemotherapy or hormone therapy [5,7,8,10,12,13,14,15,16]. However, the response rate remains low for these traditional therapies [7,8,10,14]. Crane et al. demonstrated that secondary cytoreduction results in gross residual disease in 78% of patients after completion of the surgery [14]. Goldberg et al. found that complete cytoreductive surgery in patients with recurrent LGSOC was associated with improved progression-free survival (PFS) (HR = 3.51, 95% CI = 1.72, 7.14) and overall survival (OS) (HR = 0.4, 95% CI = 0.23, 0.7) [17]. Hormone therapy in the setting of recurrence has shown to have a 9% response rate [5], while chemotherapy used in de novo or recurrent LGSOC has a response rate of 2.1–4.9% [8,10]. These traditional approaches have limited efficacy, and thus, alternative approaches have to be considered. Specific targeted therapies that target different aspects of tumor growth and development have been effective in treating other cancers and are now being investigated in LGSOC. Angiogenesis is an essential aspect of tumor growth, development, and invasion; antiangiogenic agents such as bevacizumab have been suggested as treatment modalities for various cancers. Angiogenesis relies on various cytokines and growth factors such as VEGF-A, platelet-derived growth factors, and interleukin-6 and -8. The signaling pathway of VEGF-A in angiogenesis is one of the most understood and best characterized and, thus, often used as a target to inhibit angiogenesis [7,18]. Bevacizumab is an anti-VEGF-A monoclonal antibody that binds circulating VEGFR and prevents receptor binding [18]. It is an FDA-approved antiangiogenic agent that has shown to be an effective treatment regimen for several forms of cancer, including metastatic epithelial ovarian cancer, metastatic colorectal cancer, advanced non-squamous non-small cell lung carcinoma, metastatic breast cancer, and ovarian, fallopian tube, or primary peritoneal cancer in combination with cytotoxic chemotherapy [7,15,19,20]. Bevacizumab is commonly used in the treatment of epithelial ovarian cancer in the primary setting and at the time of platinum-resistant recurrence, in combination with chemotherapy. It has been shown that bevacizumab has activity in high-grade serous ovarian cancer, with the greatest effect seen in a high-risk subgroup of patients [21]. Several studies investigating bevacizumab as a treatment for epithelial ovarian cancer have demonstrated improvements in PFS [20,21,22,23,24,25,26,27,28,29,30]. The AURELIA trial demonstrated that bevacizumab with chemotherapy in a population of platinum-resistant ovarian cancer almost doubled the PFS in the platinum-resistant population [20]. The OCTAVIA trial reported that patients treated with bevacizumab and chemotherapy followed by single-agent bevacizumab in the first-line setting had a median PFS of 23.7 months [29]. Petrillo et al. demonstrated an increase in PFS by incorporating bevacizumab as a neoadjuvant treatment with chemotherapy [30]. Oza et al. demonstrated an overall survival benefit in patients at high risk of disease progression [21]. These clinical trials, however, were largely populated by patients with high-grade ovarian cancer, and there are limited studies that have evaluated the effect of bevacizumab on the low-grade serous ovarian cancer population. The objective of this systematic review is to assess the effectiveness of bevacizumab on overall response rate, progression-free survival, and overall survival in patients with LGSOC, as well as to assess its safety in this patient population.

## 2. Materials and Methods

### 2.1. Search Strategy

This systematic review was completed following the Preferred Reporting Items for Systematic Review and Meta-Analysis (PRISMA) guidelines and was registered on Research Registry (reaseachregistry.com, Review Registry UIN: reviewregistry1698) [31]. A comprehensive search of the following databases was completed by a librarian with expertise in gynecologic oncology: Medline ALL (Medline and Epub Ahead of Print and In-Process and Other Non-Indexed Citations), Cochrane Central Register of Controlled Trials, Cochrane Database of Systematic Reviews, and Embase all from the OvidSP platform, from the inception of each database to February 2022. No language restrictions were applied, but the search was limited to human studies and adults (over 19 years) where applicable. Each search strategy comprised a combination of controlled vocabulary terms and text words, adapting the database-specific search syntax. The search strategy is available in the supplementary material (Supplementary Materials S1). In addition, ClinicalTrials.gov, International Clinical Trials Registry Platform (ICTRP), and International Standard Randomised Controlled Trial Number Registry (ISRCTN) were searched from the inception of each registry to February 2022 to identify any ongoing trials. Eligible citations were imported into Endnote X7, and duplicates were removed. Then, citations were imported to Covidence Online Systematic Review software, where additional duplicates were identified and removed. The citations were then reviewed independently by two reviewers (CL and RL).

### 2.2. Study Selection

Studies describing the use of bevacizumab on participants with low-grade serous ovarian cancer in the primary or recurrent setting were included for review by two independent reviewers (CL and RL). Studies were excluded if histology was not low-grade serous ovarian cancer or if animal or in vitro studies were completed. Research in progress, dissertations, book chapters, conferences, and review articles or commentaries were also excluded.

**Figure 1:**
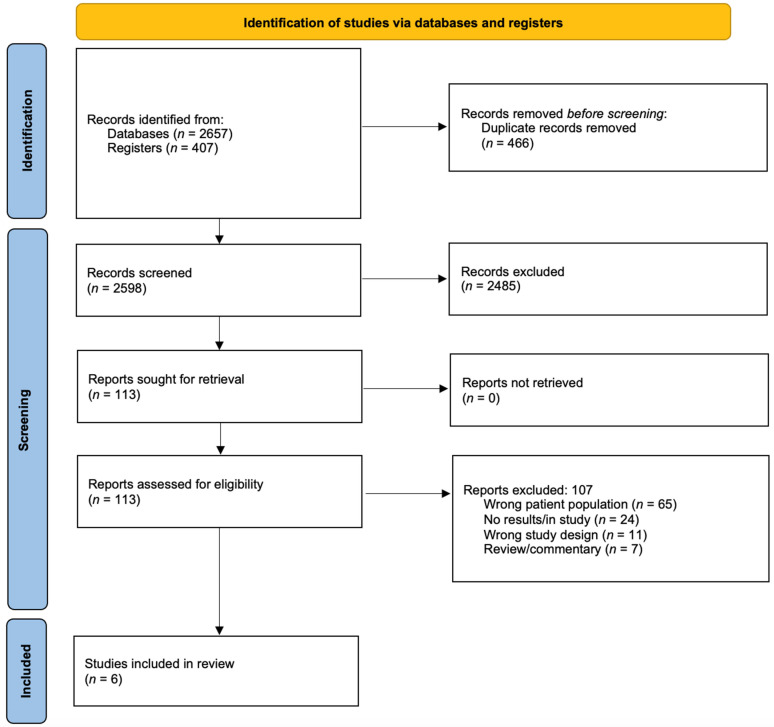
PRISMA diagram.

Two independent reviewers (CL and RL) assessed all citations in two stages (titles/abstract and full text) using a standardized electronic systematic review software (Covidence systematic review software, Veritas Health Innovation, Melbourne, Australia). Reviewers were not blinded to the journal or author. Disagreements were resolved by article review and discussion between the two reviewing authors and the principal investigator (TZ). The reason for exclusion was documented during the full-text review stage. The PRISMA figure of study selection is included in Figure 1.

### 2.3. Data Extraction

Data was extracted into a standardized template (Covidence systematic review software, Veritas Health Innovation, Melbourne, Australia) independently by two reviewers (CL and TZ). Extracted data included article title, first author, publication year, journal, country of study, aim of study, study design, number of participants, start and end date, population description, inclusion and exclusion criteria, number of participants, age of participants, cancer stage, treatment regimen, median length of follow-up, overall response rate, progression-free survival, overall survival, and adverse events. The primary outcome of interest was the overall response rate following bevacizumab treatment. The secondary outcomes of interest were the progression-free survival and overall survival of participants treated with bevacizumab and reported adverse events.

### 2.4. Critical Appraisal

All final articles included in this systematic review underwent a critical appraisal to assess the quality of the study (Table 1. Critical appraisal assessment was completed independently by two reviewers (CL and TZ). The JBI critical appraisal checklists were used for each study type (case reports [32], case series [33], and randomized control trials [34]). Since various JBI appraisal tools were used, the number of appraisal questions that had their criteria met was summarized as a fraction of the total number of questions in the appraisal tool to compare the appraisals of the various study designs included in this review. We allocated a quality rating system of high, medium, or low based on the scores. Of note, the quality rating assigned is not a cross-comparison among the different study types, but rather, it is a rating applied based on the score from the JBI appraisal tool for the individual study type. This provides an objective measure of the study quality based on the study design.

## 3. Results

### 3.1. Study Selection

In total, 3064 relevant studies were identified in our literature search, and 466 duplicates were excluded. The remaining 2598 studies were screened by title and abstract, which removed 2485 irrelevant articles. The remaining 113 studies were eligible for full-text screening. This screening excluded 107 articles for the following reasons: 65 for the wrong patient population, 24 for lack of results/study in progress, 11 for the wrong study design, and 7 for review/commentary articles. The remaining 6 studies were included in this systematic review [7,15,16,21,35,36].

### 3.2. Study Characteristics

There were six studies included in this systematic review, which are summarized in Table 2. Of the studies included in this review, there were five retrospective studies (one case report [35], one case series [36], and three cohort studies [7,15,16]) and one randomized control trial [21]. Most studies originated from the United States, except the study by Oza et al., which was completed across 11 countries [21]. All studies were published between 2006 and 2017. Five of the studies used bevacizumab treatment in the recurrent setting, and one study reported the effect of bevacizumab in the primary setting. There were a total of 1601 participants included in these studies. However, only 153 met the inclusion criteria for this study as these patients were diagnosed with LGSOC. There was a median sample size of 14.5 (range 1–80). Where cancer stage was reported, the majority were stage IIIC (*n* = 86, 56.2%); however, only four articles included cancer stage [7,21,35,36]. The treatment regimen varied significantly within and between studies; however, bevacizumab with carboplatin and paclitaxel was the most common treatment, with 35 participants (22.8%) receiving this regimen.

### 3.3. Outcomes

The results from the included studies are summarized in Table 2. The overall response rate (ORR) ranged from 8.3–100%, with a median ORR of 47.5%. Adverse events reported in each study are demonstrated in Table 1. Grisham, Rose, and Dalton only reported adverse events that resulted in the patient discontinuing bevacizumab treatment [7,15,16]. Oza et al. reported adverse events for all patients included in the study population who were treated with bevacizumab and did not report adverse events on the specific subgroup of interest (i.e., LGSOC) [21]. Thus, in total, 494 (59%) patients treated with bevacizumab had documented side effects, 18 (1.1%) of which resulted in discontinuation of treatment.

The case series by Bidus et al. demonstrated that the use of bevacizumab in well-differentiated, heavily pretreated, and recurrent LGSOC resulted in an overall response rate of 100% (*n* = 3), with 67% (*n* = 2) of participants obtaining a partial response and 33% (*n* = 1) of participants obtaining a complete response. Patients were treated with 15 mg/m^2^ of bevacizumab. Moreover, there were limited adverse effects from the treatment, and no participants stopped bevacizumab treatment due to adverse events. The most common adverse events reported were severe myalgia and fatigue, with 100% (*n* = 3) of patients experiencing both these adverse events [36]. In the 2013 case study by Rose et al., a similar response was observed. A sustained partial response was seen after cycle 25 in a participant with recurrent LGSOC treated with bevacizumab and cyclophosphamide. They also described that the participant elected to stop treatment, resulting in recurrence, which was detected by a CT scan showing increased metastasis 4 months after discontinuation of treatment, with associated abdominal pain. In this patient, re-initiation of the treatment regimen led to improvement of these symptoms. This patient reported no adverse effects, but a CT scan demonstrated experienced mesenteric artery stenosis after cycle 61, which was managed conservatively [35]. In the retrospective, single-institution study by Grisham et al., it was reported that following treatment with bevacizumab alone or in combination with chemotherapy, a partial response rate of 40% (*n* = 6) was identified, and 33% (*n* = 5) of participants studied had stable disease. The participant population was mixed with LGSOC (*n* = 10, 59%), LGS peritoneal cancer (*n* = 3, 18%), and serous borderline disease (*n* = 4, 24%). Moreover there were six different treatment regimens [bevacizumab + paclitaxel (*n* = 7, 41%), bevacizumab + oral cyclophosphamide (*n* = 3, 18%), bevacizumab + gemcitabine + carboplatin (*n* = 2, 12%), bevacizumab + gemcitabine (*n* = 2, 12%), bevacizumab alone (*n* = 2, 12%), bevacizumab + topotecan (*n* = 1, 6%)], and three different doses of bevacizumab were used [10 mg/kg (*n* = 7, 41%), 15 mg/kg (*n* = 7, 41%), 7.5 mg/kg (*n* = 3, 18%)], making it difficult to establish the effect of bevacizumab treatment alone against LGSOC. However, by analyzing the results of only those with LGSOC and LGS peritoneal cancer, the overall response rate was 55% (*n* = 6), all of which were partial responses. Stable disease was seen in three participants (27%), and progressive disease was seen in two participants (18%). Grisham et al. only reported two adverse events that led to the discontinuation of treatment: delayed wound healing (6%, *n* = 1) and small bowel fistula (6%, *n* = 1) [7]. Rose et al. (2016) identified twelve patients with LGSOC who were treated with bevacizumab at two institutions. They demonstrated an overall response rate of 8.3% in their population, which was mostly treated with bevacizumab alone (*n* = 10, 83%). One patient was initially treated with abraxane, bevacizumab, and carboplatin for six cycles, then bevacizumab alone for four cycles. The majority of participants had stable disease (*n* = 10, 83%), and none had progressive disease. Moreover, there was a long median progression-free survival, reported as 48 months. Rose et al. only reported one adverse event that led to the discontinuation of treatment, which was a GI bleed (8%, *n* = 1) [16]. Dalton et al. studied the activity of bevacizumab in patients with LGSOC of the ovary or peritoneum. This study included 45 different treatment regimens, the majority of which were bevacizumab in combination with chemotherapy. They reported an overall response rate of 47.5% (*n* = 19), including a partial response in 40% (*n* = 16) of participants and a complete response in 7.5% (*n* = 3) of participants. They reported stable disease in 30% (*n* = 12) of participants and progression of disease in 23% (*n* = 9) of participants. It should be noted that the dose and interval of treatments were not described in this study. They reported 15 adverse events that led to the discontinuation of the patient’s treatment, the most common being GI perforation (*n* = 2, 4%) and severe hypertension (*n* = 2, 4%) [15]. Oza et al., which is the only prospective randomized trial included in this review, did not identify an overall survival benefit in LGSOC patients treated with bevacizumab in addition to carboplatin and paclitaxel (50.5 months [95% CI 43.9–57.0]) compared to those treated with carboplatin and paclitaxel in the front-line setting (50.4 months [95% CI 45.6–55.2]). Oza et al. did not include all patient demographics or adverse events reported for each subgroup, i.e., LGSOC; thus, identifying adverse events in those with LGSOC treated with bevacizumab could not be completed. However, all reported adverse events from the entire study population are included in Table 3 [21].

## 4. Discussion

Low-grade serous ovarian cancer (LGSOC) is a rare gynecologic cancer that lacks effective and evidence-based treatment regimens [7,8,10,16,35,36]. It is often compared to and treated similarly to the more prevalent types of epithelial ovarian cancers, such as high-grade serous ovarian cancer, despite emerging evidence that the various histology types have distinct behavior patterns and require tailored treatments. For example, LGSOCs often have mutations in the KRAS, BRAF, or ERBB2 genes versus high-grade ovarian carcinomas, which have TP53 mutations [4]. LGSOC is also more chemoresistant than high-grade serous ovarian cancer [15]. These unique molecular profiles result in differences in disease presentation and behavior and require disease-specific therapy regimens [4,10,36]. Traditional therapies such as chemotherapy and hormone therapy have low overall response rates, about 4% [8,10] and 9% [5], respectively, and thus alternative treatment options need to be explored. Since the 1970s, when it was hypothesized that inhibiting angiogenesis may prevent tumor growth and development, researchers have been working to identify regulators of angiogenesis with the objective of improving cancer therapies. Since then, angiogenesis pathways and regulators have been identified, and some have been successfully used as therapeutic targets. Antiangiogenic agents such as bevacizumab have been identified and used as effective cancer therapies that interrupt the angiogenesis pathway that is often upregulated in tumors. Bevacizumab is an FDA-approved anti-VEGF-A antibody that prevents receptor binding and has effectively been used in various cancers, including colorectal cancer, lung cancer, and epithelial ovarian cancer [7,15,19,20]. Several studies have demonstrated that bevacizumab improves outcomes for patients with epithelial ovarian cancer [20,21,22,23,24,25,26,27,28,29,30]. However, while bevacizumab is often used in the first line and recurrent setting in high-grade serous ovarian cancer, its role in the use of LGSOC is less understood.

This systematic review, which reviewed literature studying the use of bevacizumab in patients with LGSOC, demonstrates that bevacizumab shows promise as a treatment for LGSOC. The reported overall response rate (ORR) in this population treated with bevacizumab with or without chemotherapy ranged from 8.3% to 100%, with a median ORR of 47.5%. These response rates are much higher than the 2.1–4.9% response rate of chemotherapy alone and the 9% response rate of hormone therapy [5,8,10]. Thus, although it is often overlooked, bevacizumab should be considered as part of the systemic treatment options for patients with LGSOC. Its use can be considered in front-line therapy in combination with cytotoxic chemotherapy and/or for use in the recurrent platinum-sensitive or platinum-resistant setting for patients who have not previously received bevacizumab. However, additional well-designed, prospective studies are required in order to provide more robust evidence to support these findings.

Importantly, this review demonstrates that, overall, bevacizumab is a well-tolerated medication with manageable side effects. There were some serious side effects reported, including small bowel fistula, bowel perforation, severe hypertension, and thromboembolism (Table 3). The observed adverse events are all well-documented side effects of bevacizumab, and patients should be screened for predisposing conditions that may increase the risk of these serious adverse effects [19].

While the objective of this study included analyzing the effect of bevacizumab on both primary and recurrent LGSOC, only one study (ICON 7 by Oza et al.) identified participants treated in the front-line setting [21]. The ICON 7 study, which was a randomized control trial evaluating the use of bevacizumab in newly diagnosed patients with epithelial ovarian cancer, was largely composed of patients with high-grade serous ovarian cancer but did include a planned subgroup analysis of LGSOC patients. In this subgroup, an overall survival benefit was not demonstrated for those treated with the addition of bevacizumab to carboplatin and paclitaxel. However, the sample size was small, with only 80 patients included that had LGSOC out of a total study population of 1528; of these, 31 received bevacizumab, and 49 received standard therapy [21]. Therefore, further evidence is needed to determine the role of bevacizumab in the first-line setting. At present, its role remains unclear, and additional studies are required to investigate patients treated with bevacizumab in the front-line setting.

Currently, there are many ongoing studies evaluating bevacizumab in epithelial ovarian cancer; however, many of these studies exclude those with low-grade serous carcinomas, likely due to the rarity of LGSOC in comparison to high-grade epithelial ovarian cancers and the understanding that LGSOC has a distinctive behavior that is different from high-grade epithelial ovarian cancers. Following a review of ClinicalTrials.gov, the International Clinical Trials Registry Platform (ICTRP), and the International Standard Randomised Controlled Trial Number Registry (ISRCTN), we identified current trials being conducted in the LGSOC population. The ongoing studies evaluating LGSOC-specific treatment regimens include MEK inhibitors [37,38], multi-kinase inhibitors [39,40], monoclonal antibodies [41], aromatase inhibitors [42,43], and antiprogestogens [43]. Notably, none of these trials are investigating the role of bevacizumab in LGSOC in the primary or recurrent setting or in combination with any other drug. The variety of agents being studied further highlights the ongoing challenge in treating LGSOC. Clinicians are strongly encouraged to consider enrolling patients diagnosed with LGSOC in clinical trials to help further inform the evidence to treat this rare disease. Without a collaborative effort, patients will continue to receive treatments that are considered “the current standard of care” but that have minimal efficacy and, in some treatment regimens, such as systemic chemotherapy, can have related toxicity. 

The main strength of this study is that an extensive literature search was completed by a librarian with experience and expertise in gynecologic oncology. Thus, we are confident that our search includes all relevant titles. There are some limitations to this systematic review. The first limitation is the retrospective nature of the majority of the studies included in this review. Only one study included was a randomized control trial; however, only a subgroup of the patients in this study were eligible for inclusion in our analysis. Second, there was a very small participant population and few studies that were eligible for inclusion in this review, which limits the generalizability of the results. Overall, there is limited literature on this topic, and of the existing literature, few participants are included. This is to be expected as LGSOC is a rare gynecologic malignancy. However, the lack of evidence limits the generalizability of the study outcomes and results in poor management of these patients, as we are yet to determine the optimal management guidelines. Overall, there are very few literature articles that study bevacizumab in the LGSOC population, and the studies that exist are quite heterogenous in nature, making direct comparisons of results challenging. To provide a comprehensive review of the literature that exists to date on this rare disease, we opted to include all study designs in our systematic review, including case series and as well as a subgroup from a randomized control trial. Additionally, there were many different treatment regimens used in the studies included in this review. For example, Dalton et al. included 45 different treatment regimens for 40 participants [15]. Moreover, the dose and schedule of bevacizumab were not documented in all studies. These limitations make it challenging to analyze the effect of bevacizumab on LGSOC and draw conclusions from the available data. There is an overall lack of robust data surrounding the effect of bevacizumab on LGSOC. Thus, well-designed trials are needed to determine the optimal management of LGSOC, given the small patient population.

## 5. Conclusions

In conclusion, this systematic review demonstrates that bevacizumab shows promise as a treatment option for low-grade serous ovarian cancer, though more studies are required to support these findings and help understand if the benefit is from bevacizumab alone or in combination with systemic chemotherapy. There is currently very limited data surrounding the use of bevacizumab in the LGSOC population. The results from this review, while small participant numbers and heterogeneity in the treatment regimen, did demonstrate that those who received bevacizumab had a high overall response rate from 8.3% to 100%, with a median ORR of 47.5%, contributing towards an alternative evidence-based treatment regimen for patients with LGSOC. However, given the rarity of the disease, collaborative efforts across oncology centers are needed to help elucidate the best treatment strategy for patients with LGSOC. We encourage centers with experience in the use of bevacizumab in this patient population to evaluate and publish their data in order to broaden the available literature on this topic. In addition, where possible, enrollment in patient registries and clinical trials for these patients should be considered.

## Figures and Tables

**Table 1:** Critical appraisal of studies included in this systematic review [7,15,16,21,35,36].

First Author	JBI Appraisal Tool	Criteria Met	Quality Rating *
Bidus	Case series	3/10	Low
Rose, 2013	Case report	7/8	High
Grisham	Case series	10/10	High
Oza	Randomized control trial	9/13	Medium
Rose, 2016	Case series	10/10	High
Dalton	Case series	10/10	High

* not a cross-comparison among study designs.

**Table 2:** Characteristics of studies describing bevacizumab therapy on individuals with low-grade serous ovarian cancer [7,15,16,21,35,36].

First Author	Year	Journal	Study Design	Number of Participants (*n*)	Age [Median (Range)]	Cancer	Cancer Stage (#, %)	Treatment Regimen (*n*, %)	Median F/U Time [mo, (Range)]	Response Rate (*n*, %)	Median PFS (mo)	OS
Bidus	2006	Gynecologic Oncology	Retrospective case series	3	58 (39–62)	Recurrent	IIB: 2, 66.7% IIIB: 1, 33.3%	Bev (3, 100%)	15 (15–20)	ORR: 3, 100% PR: 2, 66.7% CR: 1, 33.3%		
Rose	2013	Anti-Cancer Drugs	Retrospective case report	1	39	Recurrent	IIIC: 1, 100%	Bev + cyclophosphamide. (1, 100%)	92	ORR: 1, 100% PR: 1, 100%		
Grisham	2014	International Journal of Gynecological Cancer	Retrospective cohort study	17	47 (15–71)	Recurrent	IC: 1, 5.9% IIC: 1, 5.9% IIIA: 2, 11.8% IIIB: 1: 5.9% IIIC: 10, 58.8% IV: 2, 11.8%	Bev + paclitaxel (7, 41.2%) Bev + oral cyclophosphamide (3, 17.6%) Bev + gemcitabine + carboplatin (2, 11.8%) Bev + gemcitabine (2, 11.8%) Bev (2, 11.8%) Bev + topotecan (1, 5.9%)	5.8 (1.5–19.9)	ORR: 6, 40% PR: 6, 40% LGSOC/LGSPP ORR: 6, 54.5% PR: 6, 54.5%		5 y OS: 61.8% (95% CI, 33.6–80.8) Median OS: 102.5 mo (95% CI, 39.3-not evaluable)
Oza	2015	Lancet Oncology	Randomized control trial	Entire study: 1528 LGSOC: 80	LGSOC Bev: 47 (24–79) Control: 49 (24–71)	Primary	LGSOC IIB/IIC: n = 6, 7.5% III: n = 1, 1.2% IIIA: n = 9, 11.2% IIIB: n = 8, 10.0% IIIC: n = 53, 66.3% IV: n = 3, 3.8%	LGSOC Carboplatin + paclitaxel (49, 61%) Bev + carboplatin + paclitaxel (31, 39%)	LGSOC Bev: 55.3 (47.9–62.0) Control: 50.5 (28.2–55.1)			LGSOC Mean survival time: Bev: 50.5 mo (43.9–57.0) Control: 50.4 mo (45.6–55.2)
Rose	2016	International Journal of Gynecological Cancer	Retrospective cohort study	12	58.5 (18–75)	Recurrent		Bev (10, 83.3%) Bev + cyclophosphamide (1, 8.3%) Bev + tabraxabe + carboplatin 6 cycles, then Bev alone 4 cycles (1, 8.3%)	32	ORR: 1, 8.3% PR: 1, 8.3%	48	
Dalton	2017	Gynecologic Oncology	Retrospective cohort study	40	43.8 (20.8–80.2)	Recurrent		Bev + cyclophosphamide (8) Bev + paclitaxel (4) Bev + paclitaxel/carboplatin (4) Bev (3) Bev + docetaxel (3) Bev + aromatase inhibitor (3) Bev + sorafenib (3) Bev + gemcitabine/carboplatin (2) Bev + topotecan (2) Bev + gemcitabine (2) Bev + gemcitabine + fulvestrant (1) Bev + tamoxifen + carboplatin (1) Bev + docetaxel/carboplatin (1) Bev + pegylated liposomal doxorubicin/carboplatin (1) Bev + pegylated liposomal doxorubicin + temsirolimus (1) Bev + carboplatin (1) Bev + leuprolide acetate (1) Bev + temsirolimus (1) Bev + sorafenib + temsirolimus (1) Bev + everolimus (1) Bev + autologous vaccine (1)		ORR: 19, 47.5% PR: 16, 40% CR: 3, 7.5%	10.2 (95% CI 7.9–12.4)	34.6 (95% CI 29.5–39.7)

Abbreviations: PFS—progression-free survival, OS—overall survival, LGSOC—low-grade serous ovarian cancer, LGSPP—low-grade serous primary peritoneal cancer, bev—bevacizumab, ORR—overall response, PR—partial response, CR—complete response.

**Table 3:** Adverse events reported in patients treated with bevacizumab in each study [7,15,16,21,35,36].

Reported Adverse Event	Bidus	Rose 2013	Grisham *	Oza ^+^	Rose 2016 *	Dalton *
Overall	3 (100%)	1 (100%)	2 (11.8%)	472 (61.8%)	1 (8.3%)	15 (33.3%)
Severe myalgia	3 (100%)					
Osteodynia	1 (33.3%)					
Worsening of osteoarthritis	2 (66.7%)					
Fatigue	3 (100%)					
Mesenteric artery stenosis		1 (100%)				
Delayed wound healing			1 (5.9%)			1 (2.2%)
Small bowel fistula			1 (5.9%)	1 (0.13%)		1 (2.2%)
GI bleed					1 (8.3%)	
GI perforation				10 (01.3%)		2 (4.4%)
Severe hypertension				136 (17.8%)		2 (4.4%)
Small bowel obstruction						1 (2.2%)
Cellulitis						1 (2.2%)
GI side effects						1 (2.2%)
Hematemesis						1 (2.2%)
Acute renal failure						1 (2.2%)
Proteinuria						1 (2.2%)
Breast lymphangitis						1 (2.2%)
Epidural abscess						1 (2.2%)
Pelvic abscess						1 (2.2%)
Mucocutaneous bleeding				271 (35.4%)		
Thromboembolic event				51 (6.6%)		
Cardiac failure				1 (0.13%)		
Sarcoidosis				1 (0.13%)		
Foot fracture				1 (0.13%)		

Abbreviations: GI—gastrointestinal * Grisham, Rose 2016, and Dalton reported AEs resulting in discontinuation of treatment. ^+^ Oza reported AEs for all patients treated with bevacizumab, not only those with LGSOC.

## Data Availability

Data is contained within the article.

## Supplementary Materials

The following supporting information can be downloaded at: https://www.mdpi.com/article/10.3390/curroncol30090592/s1, Supplementary Materials S1: Search Strategy.
