# Supplementary material for: Bevacizumab Treatment for Low-Grade Serous Ovarian Cancer: A Systematic Review
Source: Curr Oncol. 2023 Sep 3;30(9):8159–71. doi: 10.3390/curroncol30090592 (PMC10528002; doi:10.3390/curroncol30090592)
Supplement: Supplementary file 1 [file curroncol-30-00592-s001.zip › curroncol-2512680-supplementary.pdf]

Ovid MEDLINE(R) ALL 1946 to August 18, 2021

#	Searches	Results	Type
1	exp Ovarian Neoplasms/	87810	Advanced
2	exp Ovary/	93677	Advanced
3	Ovarian Diseases/	6168	Advanced
4	(ovar* adj6 cancer*).mp,kw.	70580	Advanced
5	(ovar* adj6 neoplas*).mp,kw.	87846	Advanced
6	(ovar* adj6 tumo?r*).mp,kw.	33276	Advanced
7	(ovar* adj6 carcin*).mp,kw.	28516	Advanced
8	(ovar* adj6 adenocarcin*).mp,kw.	3778	Advanced
9	(ovar* adj6 adeno-carcin*).mp,kw.	15	Advanced
10	(ovar* adj6 malig*).mp,kw.	10798	Advanced
11	(ovar* adj6 metast*).mp,kw.	8616	Advanced
12	(ovar* adj6 oligo*).mp,kw.	1589	Advanced
13	(ovar* adj6 (recur* or re-cur*)).mp,kw.	6816	Advanced
14	(ovar* adj6 (reoccur* or re-occur*)).mp,kw.	4	Advanced

15	(ovar* adj6 (relaps* or re-laps*)).mp,kw.	1156	Advanced
16	(ovar* adj6 progres*).mp,kw.	4072	Advanced
17	(ovar* adj6 onco*).mp,kw.	2898	Advanced
18	HBOC.mp,kw.	1121	Advanced
19	or/1-18	216458	Advanced
20	Fallopian Tube Neoplasms/	2990	Advanced
21	Fallopian Tubes/	12439	Advanced
22	Fallopian Tube Diseases/	3221	Advanced
23	((fallopian tube? or tubal) adj6 neoplas*).mp,kw.	3285	Advanced
24	((fallopian tube? or tubal) adj6 tumo?r*).mp,kw.	609	Advanced
25	((fallopian tube? or tubal) adj6 cancer*).mp,kw.	1635	Advanced
26	((fallopian tube? or tubal) adj6 carcin*).mp,kw.	1817	Advanced
27	((fallopian tube? or tubal) adj6 adenocarcin*).mp,kw.	346	Advanced
28	((fallopian tube? or tubal) adj6 adeno-carcin*).mp,kw.	0	Advanced
29	((fallopian tube? or tubal) adj6 malign*).mp,kw.	329	Advanced
30	((fallopian tube? or tubal) adj6 metast*).mp,kw.	209	Advanced

31	((fallopian tube? or tubal) adj6 oligo*).mp,kw.	24	Advanced
32	((fallopian tube? or tubal) adj6 (recur* or re-cur*)).mp,kw.	411	Advanced
33	((fallopian tube? or tubal) adj6 (reoccur* or re-occur*)).mp,kw.	0	Advanced
34	((fallopian tube? or tubal) adj6 (relaps* or re-laps*)).mp,kw.	22	Advanced
35	((fallopian tube? or tubal) adj6 progres*).mp,kw.	96	Advanced
36	((fallopian tube? or tubal) adj6 onco*).mp,kw.	42	Advanced
37	PFTC.mp,kw.	97	Advanced
38	or/20-37	18597	Advanced
39	Peritoneal Neoplasms/	15932	Advanced
40	Peritoneum/	14697	Advanced
41	(periton* adj6 neoplas*).mp,kw.	18730	Advanced
42	(periton* adj6 cancer*).mp,kw.	7136	Advanced
43	(periton* adj6 tumo?r*).mp,kw.	6592	Advanced
44	(periton* adj6 carcin*).mp,kw.	7054	Advanced
45	(periton* adj6 adenocarcin*).mp,kw.	737	Advanced
46	(periton* adj6 adeno-carcin*).mp,kw.	1	Advanced

47	(periton* adj6 malign*).mp,kw.	3310	Advanced
48	(periton* adj6 metast*).mp,kw.	7489	Advanced
49	(periton* adj6 oligo*).mp,kw.	187	Advanced
50	(periton* adj6 (recur* or re-cur*)).mp,kw.	3713	Advanced
51	(periton* adj6 (reoccur* or re-occur*)).mp,kw.	3	Advanced
52	(periton* adj6 (relaps* or re-laps*)).mp,kw.	449	Advanced
53	(periton* adj6 progres*).mp,kw.	1028	Advanced
54	(periton* adj6 onco*).mp,kw.	426	Advanced
55	psammomacarcino*.mp,kw.	2	Advanced
56	psammoma-carcino*.mp,kw.	1	Advanced
57	or/39-56	45787	Advanced
58	19 or 38 or 57	267185	Advanced
59	"Neoplasms, Cystic, Mucinous, and Serous"/	969	Advanced
60	exp Cystadenocarcinoma/	8004	Advanced
61	Cystadenocarcinoma, Serous/	4180	Advanced
62	exp Cystadenoma/	6265	Advanced

63	Cystadenoma, Serous/	1074	Advanced
64	"Neoplasms, Glandular and Epithelial"/	6827	Advanced
65	serous*.mp,kw.	28556	Advanced
66	epithelial*.mp,kw.	397700	Advanced
67	(low adj3 grade?).mp,kw.	60305	Advanced
68	cystadeno*.mp,kw.	16388	Advanced
69	or/59-68	483826	Advanced
70	58 and 69	38211	Advanced
71	(low adj2 grade? adj5 serous*).mp,kw.	733	Advanced
72	LGS*.mp,kw.	1981	Advanced
73	or/70-72	40026	Advanced
74	Antibodies, Monoclonal, Humanized/	45825	Advanced
75	Bevacizumab/	12482	Advanced
76	bevacizumab*.mp,kw.	19788	Advanced
77	abp 215.mp,kw.	10	Advanced
78	abp215.mp,kw.	1	Advanced

79	ask b1202.mp,kw.	0	Advanced
80	askb1202.mp,kw.	0	Advanced
81	abevmy*.mp,kw.	0	Advanced
82	ainex*.mp,kw.	1	Advanced
83	altuzan*.mp,kw.	2	Advanced
84	alymsys*.mp,kw.	0	Advanced
85	avastin*.mp,kw.	1605	Advanced
86	aybintio*.mp,kw.	1	Advanced
87	bat 1706.mp,kw.	0	Advanced
88	bat1706.mp,kw.	2	Advanced
89	"bcd 021".mp,kw.	0	Advanced
90	bcd021.mp,kw.	1	Advanced
91	bevax*.mp,kw.	0	Advanced
92	bevz 92.mp,kw.	0	Advanced
93	bevz92.mp,kw.	1	Advanced
94	bi 695502.mp,kw.	1	Advanced

95	bi695502.mp,kw.	0	Advanced
96	bryxta*.mp,kw.	1	Advanced
97	chs 5217.mp,kw.	0	Advanced
98	chs5217.mp,kw.	0	Advanced
99	ct p16.mp,kw.	1	Advanced
100	ctp16.mp,kw.	0	Advanced
101	fkb 238.mp,kw.	0	Advanced
102	fkb238.mp,kw.	2	Advanced
103	hd 204.mp,kw.	2	Advanced
104	hd204.mp,kw.	0	Advanced
105	"hlx 04".mp,kw.	0	Advanced
106	hlx04.mp,kw.	2	Advanced
107	krabeva*.mp,kw.	0	Advanced
108	kyomarc*.mp,kw.	0	Advanced
109	lextemy*.mp,kw.	0	Advanced
110	"mb 02".mp,kw.	19	Advanced

111	mb02.mp,kw.	7	Advanced
112	mil 60.mp,kw.	2	Advanced
113	mil60.mp,kw.	2	Advanced
114	mvasi*.mp,kw.	9	Advanced
115	myl 14020.mp,kw.	0	Advanced
116	myl14020.mp,kw.	0	Advanced
117	myl 1402o.mp,kw.	1	Advanced
118	myl1402o.mp,kw.	0	Advanced
119	nsc 704865.mp,kw.	2	Advanced
120	nsc704865.mp,kw.	0	Advanced
121	onbevzi*.mp,kw.	0	Advanced
122	ons 1045.mp,kw.	0	Advanced
123	ons1045.mp,kw.	0	Advanced
124	ons 5010.mp,kw.	0	Advanced
125	ons5010.mp,kw.	0	Advanced
126	oyavas*.mp,kw.	0	Advanced

127	"pf 06439535".mp,kw.	5	Advanced
128	pf06439535.mp,kw.	0	Advanced
129	pf 6439535.mp,kw.	0	Advanced
130	pf6439535.mp,kw.	0	Advanced
131	ql 1101.mp,kw.	0	Advanced
132	ql1101.mp,kw.	2	Advanced
133	rg 435.mp,kw.	1	Advanced
134	rg435.mp,kw.	0	Advanced
135	rhuMAb-VEGF.mp,kw.	18	Advanced
136	ro 4876646.mp,kw.	0	Advanced
137	ro4876646.mp,kw.	0	Advanced
138	sb 8.mp,kw.	30	Advanced
139	sb8.mp,kw.	28	Advanced
140	stc 103.mp,kw.	0	Advanced
141	stc103.mp,kw.	0	Advanced
142	zirabev*.mp,kw.	3	Advanced

143	2s9zzm9q9v.rn.	12482	Advanced
144	1438851-35-4.rn.	0	Advanced
145	216974-75-3.rn.	0	Advanced
146	or/74-145	57745	Advanced
147	73 and 146	542	Advanced
148	(exp animals/ or exp animal experiment/) not ((exp animals/ or exp animal experiment/) and exp humans/)	4875846	Advanced
149	147 not 148	540	Advanced
150	limit 149 to ("all infant (birth to 23 months)" or "all child (0 to 18 years)" or "newborn infant (birth to 1 month)" or "infant (1 to 23 months)" or "preschool child (2 to 5 years)" or "child (6 to 12 years)" or "adolescent (13 to 18 years)")	8	Advanced
151	limit 149 to ("all adult (19 plus years)" or "young adult (19 to 24 years)" or "adult (19 to 44 years)" or "young adult and adult (19-24 and 19-44)" or "middle age (45 to 64 years)" or "middle aged (45 plus years)" or "all aged (65 and over)" or "aged (80 and over)")	235	Advanced
152	150 not 151	1	Advanced
153	149 not 152	539	Advanced

Ovid MEDLINE(R) ALL August 2021 to February 14, 2022

#	Searches	Results	T
1	exp Ovarian Neoplasms/	90298	Adv

2	exp Ovary/	95185	Adv
3	Ovarian Diseases/	6246	Adv
4	(ovar* adj6 cancer*).mp,kw.	73011	Adv
5	(ovar* adj6 neoplas*).mp,kw.	90095	Adv
6	(ovar* adj6 tumo?r*).mp,kw.	34035	Adv
7	(ovar* adj6 carcin*).mp,kw.	29388	Adv
8	(ovar* adj6 adenocarcin*).mp,kw.	3846	Adv
9	(ovar* adj6 adeno-carcin*).mp,kw.	15	Adv
10	(ovar* adj6 malig*).mp,kw.	11076	Adv
11	(ovar* adj6 metast*).mp,kw.	8854	Adv
12	(ovar* adj6 oligo*).mp,kw.	1614	Adv
13	(ovar* adj6 (recur* or re-cur*)).mp,kw.	7049	Adv
14	(ovar* adj6 (reoccur* or re-occur*)).mp,kw.	5	Adv
15	(ovar* adj6 (relaps* or re-laps*)).mp,kw.	1191	Adv
16	(ovar* adj6 progres*).mp,kw.	4295	Adv
17	(ovar* adj6 onco*).mp,kw.	3030	Adv
18	HBOC.mp,kw.	1165	Adv
19	or/1-18	221077	Adv
20	Fallopian Tube Neoplasms/	3046	Adv
21	Fallopian Tubes/	12561	Adv
22	Fallopian Tube Diseases/	3253	Adv
23	((fallopian tube? or tubal) adj6 neoplas*).mp,kw.	3346	Adv
24	((fallopian tube? or tubal) adj6 tumo?r*).mp,kw.	616	Adv
25	((fallopian tube? or tubal) adj6 cancer*).mp,kw.	1698	Adv
26	((fallopian tube? or tubal) adj6 carcin*).mp,kw.	1854	Adv
27	((fallopian tube? or tubal) adj6 adenocarcin*).mp,kw.	348	Adv
28	((fallopian tube? or tubal) adj6 adeno-carcin*).mp,kw.	0	Adv

29	((fallopian tube? or tubal) adj6 malign*).mp,kw.	336	Adv
30	((fallopian tube? or tubal) adj6 metast*).mp,kw.	211	Adv
31	((fallopian tube? or tubal) adj6 oligo*).mp,kw.	24	Adv
32	((fallopian tube? or tubal) adj6 (recur* or re-cur*)).mp,kw.	422	Adv
33	((fallopian tube? or tubal) adj6 (reoccur* or re-occur*)).mp,kw.	0	Adv
34	((fallopian tube? or tubal) adj6 (relaps* or re-laps*)).mp,kw.	23	Adv
35	((fallopian tube? or tubal) adj6 progres*).mp,kw.	98	Adv
36	((fallopian tube? or tubal) adj6 onco*).mp,kw.	43	Adv
37	PFTC.mp,kw.	100	Adv
38	or/20-37	18819	Adv
39	Peritoneal Neoplasms/	16418	Adv
40	Peritoneum/	14956	Adv
41	(periton* adj6 neoplas*).mp,kw.	19266	Adv
42	(periton* adj6 cancer*).mp,kw.	7425	Adv
43	(periton* adj6 tumo?r*).mp,kw.	6722	Adv
44	(periton* adj6 carcin*).mp,kw.	7254	Adv
45	(periton* adj6 adenocarcin*).mp,kw.	755	Adv
46	(periton* adj6 adeno-carcin*).mp,kw.	1	Adv
47	(periton* adj6 malign*).mp,kw.	3422	Adv
48	(periton* adj6 metast*).mp,kw.	7800	Adv
49	(periton* adj6 oligo*).mp,kw.	192	Adv
50	(periton* adj6 (recur* or re-cur*)).mp,kw.	3840	Adv
51	(periton* adj6 (reoccur* or re-occur*)).mp,kw.	3	Adv
52	(periton* adj6 (relaps* or re-laps*)).mp,kw.	460	Adv
53	(periton* adj6 progres*).mp,kw.	1074	Adv
54	(periton* adj6 onco*).mp,kw.	440	Adv
55	psammomacarcino*.mp,kw.	2	Adv

56	psammoma-carcino*.mp,kw.	1	Adv
57	or/39-56	46818	Adv
58	19 or 38 or 57	272711	Adv
59	"Neoplasms, Cystic, Mucinous, and Serous"/	1018	Adv
60	exp Cystadenocarcinoma/	8192	Adv
61	Cystadenocarcinoma, Serous/	4349	Adv
62	exp Cystadenoma/	6320	Adv
63	Cystadenoma, Serous/	1103	Adv
64	"Neoplasms, Glandular and Epithelial"/	6928	Adv
65	serous*.mp,kw.	29390	Adv
66	epithelial*.mp,kw.	408508	Adv
67	(low adj3 grade?).mp,kw.	62321	Adv
68	cystadeno*.mp,kw.	16679	Adv
69	or/59-68	497215	Adv
70	58 and 69	39425	Adv
71	(low adj2 grade? adj5 serous*).mp,kw.	765	Adv
72	LGS*.mp,kw.	2068	Adv
73	or/70-72	41316	Adv
74	Antibodies, Monoclonal, Humanized/	48862	Adv
75	Bevacizumab/	13035	Adv
76	bevacizumab*.mp,kw.	20578	Adv
77	abp 215.mp,kw.	10	Adv
78	abp215.mp,kw.	1	Adv
79	ask b1202.mp,kw.	0	Adv
80	askb1202.mp,kw.	0	Adv
81	abevmy*.mp,kw.	1	Adv
82	ainex*.mp,kw.	1	Adv

83	altuzan*.mp,kw.	2	Adv
84	alymsys*.mp,kw.	0	Adv
85	avastin*.mp,kw.	1628	Adv
86	aybintio*.mp,kw.	1	Adv
87	bat 1706.mp,kw.	0	Adv
88	bat1706.mp,kw.	2	Adv
89	"bcd 021".mp,kw.	1	Adv
90	bcd021.mp,kw.	1	Adv
91	bevax*.mp,kw.	0	Adv
92	bevz 92.mp,kw.	0	Adv
93	bevz92.mp,kw.	1	Adv
94	bi 695502.mp,kw.	2	Adv
95	bi695502.mp,kw.	0	Adv
96	bryxta*.mp,kw.	1	Adv
97	chs 5217.mp,kw.	0	Adv
98	chs5217.mp,kw.	0	Adv
99	ct p16.mp,kw.	2	Adv
100	ctp16.mp,kw.	0	Adv
101	fkb 238.mp,kw.	0	Adv
102	fkb238.mp,kw.	2	Adv
103	hd 204.mp,kw.	2	Adv
104	hd204.mp,kw.	1	Adv
105	"hlx 04".mp,kw.	0	Adv
106	hlx04.mp,kw.	2	Adv
107	krabeva*.mp,kw.	0	Adv
108	kyomarc*.mp,kw.	0	Adv
109	lextemy*.mp,kw.	1	Adv

110 "mb 02".mp,kw.	19	Adv
111 mb02.mp,kw.	8	Adv
112 mil 60.mp,kw.	2	Adv
113 mil60.mp,kw.	3	Adv
114 mvasi*.mp,kw.	11	Adv
115 myl 14020.mp,kw.	0	Adv
116 myl14020.mp,kw.	0	Adv
117 myl 1402o.mp,kw.	4	Adv
118 myl1402o.mp,kw.	0	Adv
119 nsc 704865.mp,kw.	2	Adv
120 nsc704865.mp,kw.	0	Adv
121 onbevzi*.mp,kw.	0	Adv
122 ons 1045.mp,kw.	0	Adv
123 ons1045.mp,kw.	0	Adv
124 ons 5010.mp,kw.	0	Adv
125 ons5010.mp,kw.	0	Adv
126 oyavas*.mp,kw.	0	Adv
127 "pf 06439535".mp,kw.	5	Adv
128 pf06439535.mp,kw.	0	Adv
129 pf 6439535.mp,kw.	0	Adv
130 pf6439535.mp,kw.	0	Adv
131 ql 1101.mp,kw.	0	Adv
132 ql1101.mp,kw.	2	Adv
133 rg 435.mp,kw.	1	Adv
134 rg435.mp,kw.	0	Adv
135 rhuMAb-VEGF.mp,kw.	18	Adv
136 ro 4876646.mp,kw.	0	Adv

137	ro4876646.mp,kw.	0	Adv
138	sb 8.mp,kw.	30	Adv
139	sb8.mp,kw.	30	Adv
140	stc 103.mp,kw.	0	Adv
141	stc103.mp,kw.	0	Adv
142	zirabev*.mp,kw.	3	Adv
143	2s9zzm9q9v.rn.	13035	Adv
144	1438851-35-4.rn.	0	Adv
145	216974-75-3.rn.	0	Adv
146	or/74-145	61520	Adv
147	73 and 146	588	Adv
148	(exp animals/ or exp animal experiment/) not ((exp animals/ or exp animal experiment/) and exp humans/)	4959044	Adv
149	147 not 148	586	Adv
150	limit 149 to ("all infant (birth to 23 months)" or "all child (0 to 18 years)" or "newborn infant (birth to 1 month)" or "infant (1 to 23 months)" or "preschool child (2 to 5 years)" or "child (6 to 12 years)" or "adolescent (13 to 18 years)")	10	Adv
151	limit 149 to ("all adult (19 plus years)" or "young adult (19 to 24 years)" or "adult (19 to 44 years)" or "young adult and adult (19-24 and 19-44)" or "middle age (45 to 64 years)" or "middle aged (45 plus years)" or "all aged (65 and over)" or "aged (80 and over)")	262	Adv
152	150 not 151	1	Adv
153	149 not 152	585	Adv
154	(202108* or 202109* or 20211* or 2022*).da.	1262472	Adv
155	(202108* or 202109* or 20211* or 2022*).dt.	820292	Adv
156	(202108* or 202109* or 20211* or 2022*).ed.	820025	Adv
157	(202108* or 202109* or 20211* or 2022*).ep.	636361	Adv

158	(202108* or 202109* or 20211* or 2022*).ez.	808785	Adv
159	or/154-158	1347214	Adv
160	153 and 159	71	Adv

Embase Classic +Embase 1947 to 2021 August 18

#	Searches	Results	T
1	exp ovary tumor/	162734	Adv
2	exp ovary cancer/	131380	Adv
3	exp ovary/	200850	Adv
4	ovary disease/	6272	Adv
5	(ovar* adj6 cancer*).mp,kw.	135507	Adv
6	(ovar* adj6 neoplas*).mp,kw.	16600	Adv
7	(ovar* adj6 tumo?r*).mp,kw.	66424	Adv
8	(ovar* adj6 carcin*).mp,kw.	53439	Adv
9	(ovar* adj6 adenocarcin*).mp,kw.	6717	Adv
10	(ovar* adj6 adeno-carcin*).mp,kw.	33	Adv
11	(ovar* adj6 malig*).mp,kw.	16650	Adv
12	(ovar* adj6 metast*).mp,kw.	15222	Adv
13	(ovar* adj6 oligo*).mp,kw.	2452	Adv
14	(ovar* adj6 (recur* or re-cur*)).mp,kw.	8837	Adv
15	(ovar* adj6 (reoccur* or re-occur*)).mp,kw.	8	Adv
16	(ovar* adj6 (relaps* or re-laps*)).mp,kw.	2112	Adv
17	(ovar* adj6 progres*).mp,kw.	5891	Adv
18	(ovar* adj6 onco*).mp,kw.	8677	Adv
19	HBOC.mp,kw.	1760	Adv
20	or/1-19	393165	Adv
21	uterine tube tumor/	1544	Adv

22	uterine tube carcinoma/	2758	Adv
23	exp fallopian tube/	3034	Adv
24	uterine tube disease/	2364	Adv
25	((fallopian or uterine) adj (tube? or tubal) adj6 neoplas*).mp,kw.	655	Adv
26	((fallopian or uterine) adj (tube? or tubal) adj6 tumo?r*).mp,kw.	2859	Adv
27	((fallopian or uterine) adj (tube? or tubal) adj6 cancer*).mp,kw.	3068	Adv
28	((fallopian or uterine) adj (tube? or tubal) adj6 carcino*).mp,kw.	4026	Adv
29	((fallopian or uterine) adj (tube? or tubal) adj6 adenocarcino*).mp,kw.	397	Adv
30	((fallopian or uterine) adj (tube? or tubal) adj6 adeno-carcino*).mp,kw.	1	Adv
31	((fallopian or uterine) adj (tube? or tubal) adj6 malign*).mp,kw.	440	Adv
32	((fallopian or uterine) adj (tube? or tubal) adj6 metast*).mp,kw.	298	Adv
33	((fallopian tube? or tubal) adj6 oligo*).mp,kw.	29	Adv
34	((fallopian tube? or tubal) adj6 (recur* or re-cur*)).mp,kw.	575	Adv
35	((fallopian tube? or tubal) adj6 (reoccur* or re-occur*)).mp,kw.	0	Adv
36	((fallopian tube? or tubal) adj6 (relaps* or re-laps*)).mp,kw.	51	Adv
37	((fallopian tube? or tubal) adj6 progres*).mp,kw.	136	Adv
38	((fallopian or uterine) adj (tube? or tubal) adj6 onco*).mp,kw.	101	Adv
39	PFTC.mp,kw.	145	Adv
40	or/21-39	12335	Adv
41	exp peritoneum tumor/	30948	Adv
42	exp peritoneum cancer/	22589	Adv
43	exp peritoneum/	76078	Adv
44	(periton* adj6 neoplas*).mp,kw.	2630	Adv
45	(periton* adj6 cancer*).mp,kw.	17637	Adv
46	(periton* adj6 tumo?r*).mp,kw.	15444	Adv
47	(periton* adj6 carcino*).mp,kw.	13827	Adv

48	(periton* adj6 adenocarcino*).mp,kw.	1451	Adv
49	(periton* adj6 adeno-carcino*).mp,kw.	1	Adv
50	(periton* adj6 malign*).mp,kw.	5054	Adv
51	(periton* adj6 metast*).mp,kw.	16051	Adv
52	(periton* adj6 oligo*).mp,kw.	207	Adv
53	(periton* adj6 (recur* or re-cur*)).mp,kw.	5050	Adv
54	(periton* adj6 (reoccur* or re-occur*)).mp,kw.	4	Adv
55	(periton* adj6 onco*).mp,kw.	773	Adv
56	(periton* adj6 (relaps* or re-laps*)).mp,kw.	855	Adv
57	(periton* adj6 progres*).mp,kw.	1815	Adv
58	psammomacarcino*.mp,kw.	3	Adv
59	psammoma-carcino*.mp,kw.	1	Adv
60	or/41-59	124452	Adv
61	20 or 40 or 60	505461	Adv
62	cystadenocarcinoma/	6732	Adv
63	cystadenoma/	9650	Adv
64	serous*.mp,kw.	43179	Adv
65	epithelial*.mp,kw.	512701	Adv
66	cystadeno*.mp,kw.	18770	Adv
67	(low adj3 grade?).mp,kw.	94329	Adv
68	or/62-67	646110	Adv
69	61 and 68	55799	Adv
70	(low adj2 grade? adj5 serous*).mp,kw.	1267	Adv
71	LGS*.mp,kw.	2922	Adv
72	or/69-71	58410	Adv
73	monoclonal antibody/	213312	Adv
74	bevacizumab/	62901	Adv

75	bevacizumab*.mp,kw.	64767	Adv
76	abp 215.mp,kw.	36	Adv
77	abp215.mp,kw.	1	Adv
78	ask b1202.mp,kw.	1	Adv
79	askb1202.mp,kw.	0	Adv
80	abevmy*.mp,kw.	0	Adv
81	ainex*.mp,kw.	2	Adv
82	altuzan*.mp,kw.	44	Adv
83	alymsys*.mp,kw.	1	Adv
84	avastin*.mp,kw.	10484	Adv
85	aybintio*.mp,kw.	1	Adv
86	bat 1706.mp,kw.	1	Adv
87	bat1706.mp,kw.	3	Adv
88	"bcd 021".mp,kw.	10	Adv
89	bcd021.mp,kw.	2	Adv
90	bevax*.mp,kw.	3	Adv
91	bevz 92.mp,kw.	3	Adv
92	bevz92.mp,kw.	4	Adv
93	bi 695502.mp,kw.	7	Adv
94	bi695502.mp,kw.	0	Adv
95	bryxta*.mp,kw.	1	Adv
96	chs 5217.mp,kw.	0	Adv
97	chs5217.mp,kw.	0	Adv
98	ct p16.mp,kw.	1	Adv
99	ctp16.mp,kw.	0	Adv
100	fkb 238.mp,kw.	4	Adv
101	fkb238.mp,kw.	3	Adv

102	hd 204.mp,kw.	8	Adv
103	hd204.mp,kw.	1	Adv
104	"hlx 04".mp,kw.	3	Adv
105	hlx04.mp,kw.	6	Adv
106	krabeva*.mp,kw.	1	Adv
107	kyomarc*.mp,kw.	0	Adv
108	lextemy*.mp,kw.	0	Adv
109	"mb 02".mp,kw.	23	Adv
110	mb02.mp,kw.	7	Adv
111	mil 60.mp,kw.	5	Adv
112	mil60.mp,kw.	6	Adv
113	mvasi*.mp,kw.	57	Adv
114	myl 14020.mp,kw.	0	Adv
115	myl14020.mp,kw.	0	Adv
116	myl 1402o.mp,kw.	5	Adv
117	myl1402o.mp,kw.	0	Adv
118	nsc 704865.mp,kw.	10	Adv
119	nsc704865.mp,kw.	0	Adv
120	onbevzi*.mp,kw.	0	Adv
121	ons 1045.mp,kw.	0	Adv
122	ons1045.mp,kw.	0	Adv
123	ons 5010.mp,kw.	1	Adv
124	ons5010.mp,kw.	0	Adv
125	oyavas*.mp,kw.	1	Adv
126	"pf 06439535".mp,kw.	28	Adv
127	pf06439535.mp,kw.	2	Adv
128	pf 6439535.mp,kw.	0	Adv

129 pf6439535.mp,kw.	0	Adv
130 ql 1101.mp,kw.	4	Adv
131 ql1101.mp,kw.	7	Adv
132 rg 435.mp,kw.	1	Adv
133 rg435.mp,kw.	0	Adv
134 rhuMAb-VEGF.mp,kw.	30	Adv
135 ro 4876646.mp,kw.	0	Adv
136 ro4876646.mp,kw.	0	Adv
137 sb 8.mp,kw.	28	Adv
138 sb8.mp,kw.	32	Adv
139 stc 103.mp,kw.	1	Adv
140 stc103.mp,kw.	0	Adv
141 zirabev*.mp,kw.	15	Adv
142 2s9zzm9q9v.rn.	0	Adv
143 1438851-35-4.rn.	16865	Adv
144 216974-75-3.rn.	54534	Adv
145 or/73-144	273427	Adv
146 72 and 145	2511	Adv
147 (exp animals/ or exp animal experimentation/ or nonhuman/) not ((exp animals/ or exp animal experimentation/ or nonhuman/) and exp human/)	7525247	Adv
148 146 not 147	2401	Adv
149 limit 148 to (embryo <first trimester> or infant <to one year> or child <unspecified age> or preschool child <1 to 6 years> or school child <7 to 12 years> or adolescent <13 to 17 years>)	46	Adv
150 limit 148 to (adult <18 to 64 years> or aged <65+ years>)	857	Adv
151 149 not 150	18	Adv
152 148 not 151	2383	Adv

153	limit 152 to (books or chapter or conference abstract or conference paper or "conference review")	615	Adv
154	152 not 153	1768	Adv

Embase Classic +Embase August 2021 to 2022 February 11

#	Searches	Results	T
1	exp ovary tumor/	167446	Adv
2	exp ovary cancer/	135490	Adv
3	exp ovary/	201549	Adv
4	ovary disease/	6379	Adv
5	(ovar* adj6 cancer*).mp,kw.	140552	Adv
6	(ovar* adj6 neoplas*).mp,kw.	15740	Adv
7	(ovar* adj6 tumo?r*).mp,kw.	67982	Adv
8	(ovar* adj6 carcin*).mp,kw.	54755	Adv
9	(ovar* adj6 adenocarcin*).mp,kw.	6961	Adv
10	(ovar* adj6 adeno-carcin*).mp,kw.	33	Adv
11	(ovar* adj6 malig*).mp,kw.	17165	Adv
12	(ovar* adj6 metast*).mp,kw.	15669	Adv
13	(ovar* adj6 oligo*).mp,kw.	2539	Adv
14	(ovar* adj6 (recur* or re-cur*)).mp,kw.	9286	Adv
15	(ovar* adj6 (reoccur* or re-occur*)).mp,kw.	9	Adv
16	(ovar* adj6 (relaps* or re-laps*)).mp,kw.	2209	Adv
17	(ovar* adj6 progres*).mp,kw.	6243	Adv
18	(ovar* adj6 onco*).mp,kw.	8982	Adv
19	HBOC.mp,kw.	1824	Adv
20	or/1-19	399832	Adv
21	uterine tube tumor/	1550	Adv
22	uterine tube carcinoma/	2872	Adv

23	exp fallopian tube/	3378	Adv
24	uterine tube disease/	2376	Adv
25	((fallopian or uterine) adj (tube? or tubal) adj6 neoplas*).mp,kw.	648	Adv
26	((fallopian or uterine) adj (tube? or tubal) adj6 tumo?r*).mp,kw.	2896	Adv
27	((fallopian or uterine) adj (tube? or tubal) adj6 cancer*).mp,kw.	3218	Adv
28	((fallopian or uterine) adj (tube? or tubal) adj6 carcino*).mp,kw.	4170	Adv
29	((fallopian or uterine) adj (tube? or tubal) adj6 adenocarcino*).mp,kw.	402	Adv
30	((fallopian or uterine) adj (tube? or tubal) adj6 adeno-carcino*).mp,kw.	1	Adv
31	((fallopian or uterine) adj (tube? or tubal) adj6 malign*).mp,kw.	454	Adv
32	((fallopian or uterine) adj (tube? or tubal) adj6 metast*).mp,kw.	307	Adv
33	((fallopian tube? or tubal) adj6 oligo*).mp,kw.	29	Adv
34	((fallopian tube? or tubal) adj6 (recur* or re-cur*)).mp,kw.	606	Adv
35	((fallopian tube? or tubal) adj6 (reoccur* or re-occur*)).mp,kw.	0	Adv
36	((fallopian tube? or tubal) adj6 (relaps* or re-laps*)).mp,kw.	56	Adv
37	((fallopian tube? or tubal) adj6 progres*).mp,kw.	139	Adv
38	((fallopian or uterine) adj (tube? or tubal) adj6 onco*).mp,kw.	103	Adv
39	PFTC.mp,kw.	148	Adv
40	or/21-39	12820	Adv
41	exp peritoneum tumor/	32068	Adv
42	exp peritoneum cancer/	23579	Adv
43	exp peritoneum/	76838	Adv
44	(periton* adj6 neoplas*).mp,kw.	2548	Adv
45	(periton* adj6 cancer*).mp,kw.	18457	Adv
46	(periton* adj6 tumo?r*).mp,kw.	15857	Adv
47	(periton* adj6 carcino*).mp,kw.	14339	Adv
48	(periton* adj6 adenocarcino*).mp,kw.	1537	Adv

49	(periton* adj6 adeno-carcino*).mp,kw.	1	Adv
50	(periton* adj6 malign*).mp,kw.	5246	Adv
51	(periton* adj6 metast*).mp,kw.	16780	Adv
52	(periton* adj6 oligo*).mp,kw.	222	Adv
53	(periton* adj6 (recur* or re-cur*)).mp,kw.	5254	Adv
54	(periton* adj6 (reoccur* or re-occur*)).mp,kw.	4	Adv
55	(periton* adj6 onco*).mp,kw.	818	Adv
56	(periton* adj6 (relaps* or re-laps*)).mp,kw.	881	Adv
57	(periton* adj6 progres*).mp,kw.	1927	Adv
58	psammomacarcino*.mp,kw.	3	Adv
59	psammoma-carcino*.mp,kw.	1	Adv
60	or/41-59	126853	Adv
61	20 or 40 or 60	513999	Adv
62	cystadenocarcinoma/	6991	Adv
63	cystadenoma/	9832	Adv
64	serous*.mp,kw.	44531	Adv
65	epithelial*.mp,kw.	526922	Adv
66	cystadeno*.mp,kw.	19172	Adv
67	(low adj3 grade?).mp,kw.	97572	Adv
68	or/62-67	664537	Adv
69	61 and 68	57755	Adv
70	(low adj2 grade? adj5 serous*).mp,kw.	1322	Adv
71	LGS*.mp,kw.	3029	Adv
72	or/69-71	60458	Adv
73	monoclonal antibody/	217431	Adv
74	bevacizumab/	65364	Adv
75	bevacizumab*.mp,kw.	67358	Adv

76	abp 215.mp,kw.	37	Adv
77	abp215.mp,kw.	1	Adv
78	ask b1202.mp,kw.	1	Adv
79	askb1202.mp,kw.	0	Adv
80	abevmy*.mp,kw.	1	Adv
81	ainex*.mp,kw.	2	Adv
82	altuzan*.mp,kw.	45	Adv
83	alymsys*.mp,kw.	2	Adv
84	avastin*.mp,kw.	10617	Adv
85	aybintio*.mp,kw.	1	Adv
86	bat 1706.mp,kw.	1	Adv
87	bat1706.mp,kw.	3	Adv
88	"bcd 021".mp,kw.	13	Adv
89	bcd021.mp,kw.	2	Adv
90	bevax*.mp,kw.	3	Adv
91	bevz 92.mp,kw.	3	Adv
92	bevz92.mp,kw.	4	Adv
93	bi 695502.mp,kw.	7	Adv
94	bi695502.mp,kw.	0	Adv
95	bryxta*.mp,kw.	2	Adv
96	chs 5217.mp,kw.	0	Adv
97	chs5217.mp,kw.	0	Adv
98	ct p16.mp,kw.	1	Adv
99	ctp16.mp,kw.	0	Adv
100	fkb 238.mp,kw.	5	Adv
101	fkb238.mp,kw.	4	Adv
102	hd 204.mp,kw.	8	Adv

103	hd204.mp,kw.	3	Adv
104	"hlx 04".mp,kw.	4	Adv
105	hlx04.mp,kw.	6	Adv
106	krabeva*.mp,kw.	1	Adv
107	kyomarc*.mp,kw.	0	Adv
108	lextemy*.mp,kw.	1	Adv
109	"mb 02".mp,kw.	25	Adv
110	mb02.mp,kw.	10	Adv
111	mil 60.mp,kw.	6	Adv
112	mil60.mp,kw.	8	Adv
113	mvasi*.mp,kw.	66	Adv
114	myl 14020.mp,kw.	0	Adv
115	myl14020.mp,kw.	0	Adv
116	myl 1402o.mp,kw.	8	Adv
117	myl1402o.mp,kw.	0	Adv
118	nsc 704865.mp,kw.	10	Adv
119	nsc704865.mp,kw.	0	Adv
120	onbevzi*.mp,kw.	0	Adv
121	ons 1045.mp,kw.	0	Adv
122	ons1045.mp,kw.	0	Adv
123	ons 5010.mp,kw.	3	Adv
124	ons5010.mp,kw.	0	Adv
125	oyavas*.mp,kw.	2	Adv
126	"pf 06439535".mp,kw.	28	Adv
127	pf06439535.mp,kw.	2	Adv
128	pf 6439535.mp,kw.	0	Adv
129	pf6439535.mp,kw.	0	Adv

130	ql 1101.mp,kw.	6	Adv
131	ql1101.mp,kw.	7	Adv
132	rg 435.mp,kw.	1	Adv
133	rg435.mp,kw.	0	Adv
134	rhuMAb-VEGF.mp,kw.	30	Adv
135	ro 4876646.mp,kw.	0	Adv
136	ro4876646.mp,kw.	0	Adv
137	sb 8.mp,kw.	32	Adv
138	sb8.mp,kw.	34	Adv
139	stc 103.mp,kw.	1	Adv
140	stc103.mp,kw.	0	Adv
141	zirabev*.mp,kw.	18	Adv
142	2s9zzm9q9v.rn.	0	Adv
143	1438851-35-4.rn.	19583	Adv
144	216974-75-3.rn.	57039	Adv
145	or/73-144	280061	Adv
146	72 and 145	2650	Adv
147	(exp animals/ or exp animal experimentation/ or nonhuman/) not ((exp animals/ or exp animal experimentation/ or nonhuman/) and exp human/)	7639233	Adv
148	146 not 147	2537	Adv
149	limit 148 to (embryo <first trimester> or infant <to one year> or child <unspecified age> or preschool child <1 to 6 years> or school child <7 to 12 years> or adolescent <13 to 17 years>)	46	Adv
150	limit 148 to (adult <18 to 64 years> or aged <65+ years>)	949	Adv
151	149 not 150	18	Adv
152	148 not 151	2519	Adv

153	limit 152 to (books or chapter or conference abstract or conference paper or "conference review")	670	Adv
154	152 not 153	1849	Adv
155	(202108* or 202109* or 20211* or 2022*).dc.	1493368	Adv
156	(20213* or 20214* or 20215* or 2022*).em.	1766380	Adv
157	or/155-156	1766380	Adv
158	154 and 157	134	Adv

Cochrane Database of Systematic Reviews August 19, 2021

#	Searches	Results	T
1	(ovar* adj6 cancer*).mp,kw.	161	Adv
2	(ovar* adj6 neoplas*).mp,kw.	57	Adv
3	(ovar* adj6 tumo?r*).mp,kw.	72	Adv
4	(ovar* adj6 carcin*).mp,kw.	35	Adv
5	(ovar* adj6 adenocarcin*).mp,kw.	2	Adv
6	(ovar* adj6 adeno-carcin*).mp,kw.	0	Adv
7	(ovar* adj6 malig*).mp,kw.	46	Adv
8	(ovar* adj6 metast*).mp,kw.	16	Adv
9	(ovar* adj6 oligo*).mp,kw.	17	Adv
10	(ovar* adj6 (recur* or re-cur*)).mp,kw.	39	Adv
11	(ovar* adj6 (reoccur* or re-occur*)).mp,kw.	0	Adv
12	(ovar* adj6 (relaps* or re-laps*)).mp,kw.	13	Adv
13	(ovar* adj6 progres*).mp,kw.	17	Adv
14	(ovar* adj6 onco*).mp,kw.	3	Adv
15	HBOC.mp,kw.	5	Adv
16	or/1-15	211	Adv

17	((fallopian tube? or tubal) adj6 neoplas*).mp,kw.	9	Adv
18	((fallopian tube? or tubal) adj6 tumo?r*).mp,kw.	7	Adv
19	((fallopian tube? or tubal) adj6 cancer*).mp,kw.	19	Adv
20	((fallopian tube? or tubal) adj6 carcin*).mp,kw.	6	Adv
21	((fallopian tube? or tubal) adj6 adenocarcin*).mp,kw.	0	Adv
22	((fallopian tube? or tubal) adj6 adeno-carcin*).mp,kw.	0	Adv
23	((fallopian tube? or tubal) adj6 malign*).mp,kw.	5	Adv
24	((fallopian tube? or tubal) adj6 metast*).mp,kw.	1	Adv
25	((fallopian tube? or tubal) adj6 oligo*).mp,kw.	0	Adv
26	((fallopian tube? or tubal) adj6 (recur* or re-cur*)).mp,kw.	4	Adv
27	((fallopian tube? or tubal) adj6 (reoccur* or re-occur*)).mp,kw.	0	Adv
28	((fallopian tube? or tubal) adj6 (relaps* or re-laps*)).mp,kw.	2	Adv
29	((fallopian tube? or tubal) adj6 progres*).mp,kw.	0	Adv
30	((fallopian tube? or tubal) adj6 onco*).mp,kw.	0	Adv
31	PFTC.mp,kw.	0	Adv
32	or/17-31	27	Adv
33	(periton* adj6 neoplas*).mp,kw.	9	Adv
34	(periton* adj6 cancer*).mp,kw.	38	Adv
35	(periton* adj6 tumo?r*).mp,kw.	17	Adv
36	(periton* adj6 carcin*).mp,kw.	27	Adv
37	(periton* adj6 adenocarcin*).mp,kw.	0	Adv
38	(periton* adj6 adeno-carcin*).mp,kw.	0	Adv
39	(periton* adj6 malign*).mp,kw.	10	Adv
40	(periton* adj6 metast*).mp,kw.	13	Adv
41	(periton* adj6 oligo*).mp,kw.	0	Adv
42	(periton* adj6 (recur* or re-cur*)).mp,kw.	21	Adv
43	(periton* adj6 (reoccur* or re-occur*)).mp,kw.	2	Adv

44	(periton* adj6 (relaps* or re-laps*)).mp,kw.	7	Adv
45	(periton* adj6 progres*).mp,kw.	5	Adv
46	(periton* adj6 onco*).mp,kw.	1	Adv
47	psammomacarcino*.mp,kw.	0	Adv
48	psammoma-carcino*.mp,kw.	0	Adv
49	or/33-48	92	Adv
50	16 or 32 or 49	264	Adv
51	serous*.mp,kw.	95	Adv
52	epithelial*.mp,kw.	533	Adv
53	(low adj3 grade?).mp,kw.	1054	Adv
54	cystadeno*.mp,kw.	9	Adv
55	or/51-54	1539	Adv
56	50 and 55	114	Adv
57	(low adj2 grade? adj5 serous*).mp,kw.	1	Adv
58	LGS*.mp,kw.	4	Adv
59	or/56-58	118	Adv
60	bevacizumab*.mp,kw.	105	Adv
61	abp 215.mp,kw.	1	Adv
62	abp215.mp,kw.	0	Adv
63	ask b1202.mp,kw.	0	Adv
64	askb1202.mp,kw.	0	Adv
65	abevmy*.mp,kw.	0	Adv
66	ainex*.mp,kw.	0	Adv
67	altuzan*.mp,kw.	2	Adv
68	alymsys*.mp,kw.	0	Adv
69	avastin*.mp,kw.	30	Adv
70	aybintio*.mp,kw.	0	Adv

71	bat 1706.mp,kw.	1	Adv
72	bat1706.mp,kw.	0	Adv
73	"bcd 021".mp,kw.	1	Adv
74	bcd021.mp,kw.	0	Adv
75	bevax*.mp,kw.	0	Adv
76	bevz 92.mp,kw.	0	Adv
77	bevz92.mp,kw.	0	Adv
78	bi 695502.mp,kw.	0	Adv
79	bi695502.mp,kw.	0	Adv
80	bryxta*.mp,kw.	0	Adv
81	chs 5217.mp,kw.	0	Adv
82	chs5217.mp,kw.	0	Adv
83	ct p16.mp,kw.	0	Adv
84	ctp16.mp,kw.	0	Adv
85	fkb 238.mp,kw.	0	Adv
86	fkb238.mp,kw.	0	Adv
87	hd 204.mp,kw.	0	Adv
88	hd204.mp,kw.	0	Adv
89	"hlx 04".mp,kw.	0	Adv
90	hlx04.mp,kw.	1	Adv
91	krabeva*.mp,kw.	0	Adv
92	kyomarc*.mp,kw.	0	Adv
93	lextemy*.mp,kw.	0	Adv
94	"mb 02".mp,kw.	0	Adv
95	mb02.mp,kw.	0	Adv
96	mil 60.mp,kw.	0	Adv
97	mil60.mp,kw.	0	Adv

98	mvasi*.mp,kw.	0	Adv
99	myl 14020.mp,kw.	0	Adv
100	myl14020.mp,kw.	0	Adv
101	myl 1402o.mp,kw.	0	Adv
102	myl1402o.mp,kw.	0	Adv
103	nsc 704865.mp,kw.	1	Adv
104	nsc704865.mp,kw.	1	Adv
105	onbevzi*.mp,kw.	0	Adv
106	ons 1045.mp,kw.	0	Adv
107	ons1045.mp,kw.	0	Adv
108	ons 5010.mp,kw.	0	Adv
109	ons5010.mp,kw.	0	Adv
110	oyavas*.mp,kw.	0	Adv
111	"pf 06439535".mp,kw.	1	Adv
112	pf06439535.mp,kw.	0	Adv
113	pf 6439535.mp,kw.	0	Adv
114	pf6439535.mp,kw.	0	Adv
115	ql 1101.mp,kw.	0	Adv
116	ql1101.mp,kw.	0	Adv
117	rg 435.mp,kw.	0	Adv
118	rg435.mp,kw.	0	Adv
119	rhuMAb-VEGF.mp,kw.	1	Adv
120	ro 4876646.mp,kw.	0	Adv
121	ro4876646.mp,kw.	0	Adv
122	sb 8.mp,kw.	1	Adv
123	sb8.mp,kw.	0	Adv
124	stc 103.mp,kw.	0	Adv

125	stc103.mp,kw.	0	Adv
126	zirabev*.mp,kw.	0	Adv
127	or/60-126	107	Adv
128	59 and 127	15	Adv
129	limit 128 to protocols	4	Adv
130	128 not 129	11	Adv

Cochrane Database of Systematic Reviews February 9, 2022

#	Searches	Results	T
1	(ovar* adj6 cancer*).mp,kw.	168	Adv
2	(ovar* adj6 neoplas*).mp,kw.	60	Adv
3	(ovar* adj6 tumo?r*).mp,kw.	75	Adv
4	(ovar* adj6 carcin*).mp,kw.	37	Adv
5	(ovar* adj6 adenocarcin*).mp,kw.	2	Adv
6	(ovar* adj6 adeno-carcin*).mp,kw.	0	Adv
7	(ovar* adj6 malig*).mp,kw.	47	Adv
8	(ovar* adj6 metast*).mp,kw.	18	Adv
9	(ovar* adj6 oligo*).mp,kw.	15	Adv
10	(ovar* adj6 (recur* or re-cur*)).mp,kw.	40	Adv
11	(ovar* adj6 (reoccur* or re-occur*)).mp,kw.	0	Adv
12	(ovar* adj6 (relaps* or re-laps*)).mp,kw.	14	Adv
13	(ovar* adj6 progres*).mp,kw.	17	Adv
14	(ovar* adj6 onco*).mp,kw.	3	Adv
15	HBOC.mp,kw.	5	Adv
16	or/1-15	216	Adv
17	((fallopian tube? or tubal) adj6 neoplas*).mp,kw.	9	Adv
18	((fallopian tube? or tubal) adj6 tumo?r*).mp,kw.	7	Adv
19	((fallopian tube? or tubal) adj6 cancer*).mp,kw.	20	Adv

20	((fallopian tube? or tubal) adj6 carcin*).mp,kw.	7	Adv[illegible]
21	((fallopian tube? or tubal) adj6 adenocarcin*).mp,kw.	0	Adv[illegible]
22	((fallopian tube? or tubal) adj6 adeno-carcin*).mp,kw.	0	Adv[illegible]
23	((fallopian tube? or tubal) adj6 malign*).mp,kw.	5	Adv[illegible]
24	((fallopian tube? or tubal) adj6 metast*).mp,kw.	1	Adv[illegible]
25	((fallopian tube? or tubal) adj6 oligo*).mp,kw.	0	Adv[illegible]
26	((fallopian tube? or tubal) adj6 (recur* or re-cur*)).mp,kw.	4	Adv[illegible]
27	((fallopian tube? or tubal) adj6 (reoccur* or re-occur*)).mp,kw.	0	Adv[illegible]
28	((fallopian tube? or tubal) adj6 (relaps* or re-laps*)).mp,kw.	2	Adv[illegible]
29	((fallopian tube? or tubal) adj6 progres*).mp,kw.	0	Adv[illegible]
30	((fallopian tube? or tubal) adj6 onco*).mp,kw.	0	Adv[illegible]
31	PFTC.mp,kw.	0	Adv[illegible]
32	or/17-31	28	Adv[illegible]
33	(periton* adj6 neoplas*).mp,kw.	9	Adv[illegible]
34	(periton* adj6 cancer*).mp,kw.	39	Adv[illegible]
35	(periton* adj6 tumo?r*).mp,kw.	16	Adv[illegible]
36	(periton* adj6 carcin*).mp,kw.	24	Adv[illegible]
37	(periton* adj6 adenocarcin*).mp,kw.	0	Adv[illegible]
38	(periton* adj6 adeno-carcin*).mp,kw.	0	Adv[illegible]
39	(periton* adj6 malign*).mp,kw.	11	Adv[illegible]
40	(periton* adj6 metast*).mp,kw.	14	Adv[illegible]
41	(periton* adj6 oligo*).mp,kw.	0	Adv[illegible]
42	(periton* adj6 (recur* or re-cur*)).mp,kw.	21	Adv[illegible]
43	(periton* adj6 (reoccur* or re-occur*)).mp,kw.	2	Adv[illegible]
44	(periton* adj6 (relaps* or re-laps*)).mp,kw.	7	Adv[illegible]
45	(periton* adj6 progres*).mp,kw.	6	Adv[illegible]
46	(periton* adj6 onco*).mp,kw.	1	Adv[illegible]

47	psammomacarcino*.mp,kw.	0	Adv
48	psammoma-carcino*.mp,kw.	0	Adv
49	or/33-48	91	Adv
50	16 or 32 or 49	266	Adv
51	serous*.mp,kw.	97	Adv
52	epithelial*.mp,kw.	551	Adv
53	(low adj3 grade?).mp,kw.	1085	Adv
54	cystadeno*.mp,kw.	9	Adv
55	or/51-54	1588	Adv
56	50 and 55	114	Adv
57	(low adj2 grade? adj5 serous*).mp,kw.	3	Adv
58	LGS*.mp,kw.	4	Adv
59	or/56-58	118	Adv
60	bevacizumab*.mp,kw.	109	Adv
61	abp 215.mp,kw.	1	Adv
62	abp215.mp,kw.	0	Adv
63	ask b1202.mp,kw.	0	Adv
64	askb1202.mp,kw.	0	Adv
65	abevmy*.mp,kw.	0	Adv
66	ainex*.mp,kw.	0	Adv
67	altuzan*.mp,kw.	2	Adv
68	alymsys*.mp,kw.	0	Adv
69	avastin*.mp,kw.	32	Adv
70	aybintio*.mp,kw.	0	Adv
71	bat 1706.mp,kw.	1	Adv
72	bat1706.mp,kw.	0	Adv
73	"bcd 021".mp,kw.	1	Adv

74	bcd021.mp,kw.	0	Adv
75	bevax*.mp,kw.	0	Adv
76	bevz 92.mp,kw.	0	Adv
77	bevz92.mp,kw.	0	Adv
78	bi 695502.mp,kw.	0	Adv
79	bi695502.mp,kw.	0	Adv
80	bryxta*.mp,kw.	0	Adv
81	chs 5217.mp,kw.	0	Adv
82	chs5217.mp,kw.	0	Adv
83	ct p16.mp,kw.	0	Adv
84	ctp16.mp,kw.	0	Adv
85	fkb 238.mp,kw.	0	Adv
86	fkb238.mp,kw.	0	Adv
87	hd 204.mp,kw.	0	Adv
88	hd204.mp,kw.	0	Adv
89	"hlx 04".mp,kw.	0	Adv
90	hlx04.mp,kw.	1	Adv
91	krabeva*.mp,kw.	0	Adv
92	kyomarc*.mp,kw.	0	Adv
93	lextemy*.mp,kw.	0	Adv
94	"mb 02".mp,kw.	0	Adv
95	mb02.mp,kw.	0	Adv
96	mil 60.mp,kw.	0	Adv
97	mil60.mp,kw.	0	Adv
98	mvasi*.mp,kw.	0	Adv
99	myl 14020.mp,kw.	0	Adv
100	myl14020.mp,kw.	0	Adv

101 myl 1402o.mp,kw.	0	Adv
102 myl1402o.mp,kw.	0	Adv
103 nsc 704865.mp,kw.	1	Adv
104 nsc704865.mp,kw.	1	Adv
105 onbevzi*.mp,kw.	0	Adv
106 ons 1045.mp,kw.	0	Adv
107 ons1045.mp,kw.	0	Adv
108 ons 5010.mp,kw.	0	Adv
109 ons5010.mp,kw.	0	Adv
110 oyavas*.mp,kw.	0	Adv
111 "pf 06439535".mp,kw.	1	Adv
112 pf06439535.mp,kw.	0	Adv
113 pf 6439535.mp,kw.	0	Adv
114 pf6439535.mp,kw.	0	Adv
115 ql 1101.mp,kw.	0	Adv
116 ql1101.mp,kw.	0	Adv
117 rg 435.mp,kw.	0	Adv
118 rg435.mp,kw.	0	Adv
119 rhuMAb-VEGF.mp,kw.	1	Adv
120 ro 4876646.mp,kw.	0	Adv
121 ro4876646.mp,kw.	0	Adv
122 sb 8.mp,kw.	1	Adv
123 sb8.mp,kw.	0	Adv
124 stc 103.mp,kw.	0	Adv
125 stc103.mp,kw.	0	Adv
126 zirabev*.mp,kw.	0	Adv
127 or/60-126	111	Adv

128	59 and 127	15	Adv
129	limit 128 to protocols	4	Adv
130	128 not 129	11	Adv
131	deduped from original search	0	Adv

Cochrane Central Register of Controlled Trials August 19, 2021

#	Searches	Results	T
1	exp Ovarian Neoplasms/	2012	Adv
2	exp Ovary/	1105	Adv
3	Ovarian Diseases/	95	Adv
4	(ovar* adj6 cancer*).mp,kw.	7285	Adv
5	(ovar* adj6 neoplas*).mp,kw.	2292	Adv
6	(ovar* adj6 tumo?r*).mp,kw.	974	Adv
7	(ovar* adj6 carcin*).mp,kw.	2085	Adv
8	(ovar* adj6 adenocarcin*).mp,kw.	246	Adv
9	(ovar* adj6 adeno-carcin*).mp,kw.	0	Adv
10	(ovar* adj6 malig*).mp,kw.	434	Adv
11	(ovar* adj6 metast*).mp,kw.	371	Adv
12	(ovar* adj6 oligo*).mp,kw.	223	Adv
13	(ovar* adj6 (recur* or re-cur*)).mp,kw.	1478	Adv
14	(ovar* adj6 (reoccur* or re-occur*)).mp,kw.	0	Adv
15	(ovar* adj6 (relaps* or re-laps*)).mp,kw.	489	Adv
16	(ovar* adj6 progres*).mp,kw.	628	Adv
17	(ovar* adj6 onco*).mp,kw.	539	Adv
18	HBOC.mp,kw.	116	Adv
19	or/1-18	10046	Adv

20	Fallopian Tube Neoplasms/	250	Adv
21	Fallopian Tubes/	155	Adv
22	Fallopian Tube Diseases/	72	Adv
23	((fallopian tube? or tubal) adj6 neoplas*).mp,kw.	261	Adv
24	((fallopian tube? or tubal) adj6 tumo?r*).mp,kw.	37	Adv
25	((fallopian tube? or tubal) adj6 cancer*).mp,kw.	851	Adv
26	((fallopian tube? or tubal) adj6 carcin*).mp,kw.	298	Adv
27	((fallopian tube? or tubal) adj6 adenocarcin*).mp,kw.	36	Adv
28	((fallopian tube? or tubal) adj6 adeno-carcin*).mp,kw.	0	Adv
29	((fallopian tube? or tubal) adj6 malign*).mp,kw.	19	Adv
30	((fallopian tube? or tubal) adj6 metast*).mp,kw.	14	Adv
31	((fallopian tube? or tubal) adj6 oligo*).mp,kw.	6	Adv
32	((fallopian tube? or tubal) adj6 (recur* or re-cur*)).mp,kw.	228	Adv
33	((fallopian tube? or tubal) adj6 (reoccur* or re-occur*)).mp,kw.	0	Adv
34	((fallopian tube? or tubal) adj6 (relaps* or re-laps*)).mp,kw.	54	Adv
35	((fallopian tube? or tubal) adj6 progres*).mp,kw.	35	Adv
36	((fallopian tube? or tubal) adj6 onco*).mp,kw.	34	Adv
37	PFTC.mp,kw.	5	Adv
38	or/20-37	1302	Adv
39	Peritoneal Neoplasms/	349	Adv
40	Peritoneum/	301	Adv
41	(periton* adj6 neoplas*).mp,kw.	446	Adv
42	(periton* adj6 cancer*).mp,kw.	1696	Adv
43	(periton* adj6 tumo?r*).mp,kw.	375	Adv
44	(periton* adj6 carcin*).mp,kw.	838	Adv
45	(periton* adj6 adenocarcin*).mp,kw.	168	Adv
46	(periton* adj6 adeno-carcin*).mp,kw.	1	Adv

47	(periton* adj6 malign*).mp,kw.	191	Adv
48	(periton* adj6 metast*).mp,kw.	674	Adv
49	(periton* adj6 oligo*).mp,kw.	9	Adv
50	(periton* adj6 (recur* or re-cur*)).mp,kw.	590	Adv
51	(periton* adj6 (reoccur* or re-occur*)).mp,kw.	0	Adv
52	(periton* adj6 (relaps* or re-laps*)).mp,kw.	124	Adv
53	(periton* adj6 progres*).mp,kw.	220	Adv
54	(periton* adj6 onco*).mp,kw.	103	Adv
55	psammomacarcino*.mp,kw.	0	Adv
56	psammoma-carcino*.mp,kw.	0	Adv
57	or/39-56	3107	Adv
58	19 or 38 or 57	12020	Adv
59	"Neoplasms, Cystic, Mucinous, and Serous"/	11	Adv
60	exp Cystadenocarcinoma/	117	Adv
61	Cystadenocarcinoma, Serous/	94	Adv
62	exp Cystadenoma/	7	Adv
63	Cystadenoma, Serous/	2	Adv
64	"Neoplasms, Glandular and Epithelial"/	236	Adv
65	serous*.mp,kw.	1497	Adv
66	epithelial*.mp,kw.	9034	Adv
67	(low adj3 grade?).mp,kw.	4240	Adv
68	cystadeno*.mp,kw.	234	Adv
69	or/59-68	14354	Adv
70	58 and 69	2635	Adv
71	(low adj2 grade? adj5 serous*).mp,kw.	43	Adv
72	LGS*.mp,kw.	211	Adv
73	or/70-72	2831	Adv

74	Antibodies, Monoclonal, Humanized/	4122	Adv
75	Bevacizumab/	2030	Adv
76	bevacizumab*.mp,kw.	7011	Adv
77	abp 215.mp,kw.	19	Adv
78	abp215.mp,kw.	0	Adv
79	ask b1202.mp,kw.	0	Adv
80	askb1202.mp,kw.	1	Adv
81	abevmy*.mp,kw.	0	Adv
82	ainex*.mp,kw.	0	Adv
83	altuzan*.mp,kw.	1	Adv
84	alymsys*.mp,kw.	0	Adv
85	avastin*.mp,kw.	909	Adv
86	aybintio*.mp,kw.	0	Adv
87	bat 1706.mp,kw.	0	Adv
88	bat1706.mp,kw.	9	Adv
89	"bcd 021".mp,kw.	5	Adv
90	bcd021.mp,kw.	0	Adv
91	bevax*.mp,kw.	3	Adv
92	bevz 92.mp,kw.	0	Adv
93	bevz92.mp,kw.	7	Adv
94	bi 695502.mp,kw.	6	Adv
95	bi695502.mp,kw.	0	Adv
96	bryxta*.mp,kw.	0	Adv
97	chs 5217.mp,kw.	0	Adv
98	chs5217.mp,kw.	0	Adv
99	ct p16.mp,kw.	7	Adv
100	ctp16.mp,kw.	0	Adv

101	fkb 238.mp,kw.	0	Adv
102	fkb238.mp,kw.	3	Adv
103	hd 204.mp,kw.	1	Adv
104	hd204.mp,kw.	5	Adv
105	"hlx 04".mp,kw.	0	Adv
106	hlx04.mp,kw.	13	Adv
107	krabeva*.mp,kw.	0	Adv
108	kyomarc*.mp,kw.	0	Adv
109	lextemy*.mp,kw.	0	Adv
110	"mb 02".mp,kw.	1	Adv
111	mb02.mp,kw.	14	Adv
112	mil 60.mp,kw.	0	Adv
113	mil60.mp,kw.	3	Adv
114	mvasi*.mp,kw.	4	Adv
115	myl 14020.mp,kw.	0	Adv
116	myl14020.mp,kw.	0	Adv
117	myl 1402o.mp,kw.	8	Adv
118	myl1402o.mp,kw.	0	Adv
119	nsc 704865.mp,kw.	33	Adv
120	nsc704865.mp,kw.	1	Adv
121	onbevzi*.mp,kw.	0	Adv
122	ons 1045.mp,kw.	0	Adv
123	ons1045.mp,kw.	0	Adv
124	ons 5010.mp,kw.	1	Adv
125	ons5010.mp,kw.	0	Adv
126	oyavas*.mp,kw.	0	Adv
127	"pf 06439535".mp,kw.	13	Adv

128	pf06439535.mp,kw.	0	Adv
129	pf 6439535.mp,kw.	0	Adv
130	pf6439535.mp,kw.	0	Adv
131	ql 1101.mp,kw.	0	Adv
132	ql1101.mp,kw.	6	Adv
133	rg 435.mp,kw.	1	Adv
134	rg435.mp,kw.	0	Adv
135	rhuMAb-VEGF.mp,kw.	69	Adv
136	ro 4876646.mp,kw.	9	Adv
137	ro4876646.mp,kw.	77	Adv
138	sb 8.mp,kw.	6	Adv
139	sb8.mp,kw.	7	Adv
140	stc 103.mp,kw.	0	Adv
141	stc103.mp,kw.	0	Adv
142	zirabev*.mp,kw.	2	Adv
143	or/74-142	10603	Adv
144	73 and 143	292	Adv
145	(abstract or book or book article or book or book note or "book review" or book series article or book series article in press or book series chapter or book series conference paper or book series letter or "book series review" or book series short survey or chapter or conference abstract or conference proceeding or "conference review" or journal conference abstract or "journal conference review").pt.	197832	Adv
146	conferenc*.so.	41322	Adv
147	145 or 146	199734	Adv
148	144 not 147	206	Adv

Cochrane Central Register of Controlled Trials January 2021 to January 2022

#	Searches	Results	T
1	exp Ovarian Neoplasms/	2062	Adv
2	exp Ovary/	1125	Adv
3	Ovarian Diseases/	96	Adv
4	(ovar* adj6 cancer*).mp,kw.	7513	Adv
5	(ovar* adj6 neoplas*).mp,kw.	2342	Adv
6	(ovar* adj6 tumo?r*).mp,kw.	993	Adv
7	(ovar* adj6 carcin*).mp,kw.	2146	Adv
8	(ovar* adj6 adenocarcin*).mp,kw.	248	Adv
9	(ovar* adj6 adeno-carcin*).mp,kw.	0	Adv
10	(ovar* adj6 malig*).mp,kw.	446	Adv
11	(ovar* adj6 metast*).mp,kw.	386	Adv
12	(ovar* adj6 oligo*).mp,kw.	232	Adv
13	(ovar* adj6 (recur* or re-cur*)).mp,kw.	1543	Adv
14	(ovar* adj6 (reoccur* or re-occur*)).mp,kw.	0	Adv
15	(ovar* adj6 (relaps* or re-laps*)).mp,kw.	513	Adv
16	(ovar* adj6 progres*).mp,kw.	669	Adv
17	(ovar* adj6 onco*).mp,kw.	548	Adv
18	HBOC.mp,kw.	118	Adv
19	or/1-18	10326	Adv
20	Fallopian Tube Neoplasms/	256	Adv
21	Fallopian Tubes/	156	Adv
22	Fallopian Tube Diseases/	72	Adv
23	((fallopian tube? or tubal) adj6 neoplas*).mp,kw.	268	Adv
24	((fallopian tube? or tubal) adj6 tumo?r*).mp,kw.	39	Adv
25	((fallopian tube? or tubal) adj6 cancer*).mp,kw.	894	Adv

26	((fallopian tube? or tubal) adj6 carcin*).mp,kw.	306	Adv
27	((fallopian tube? or tubal) adj6 adenocarcin*).mp,kw.	37	Adv
28	((fallopian tube? or tubal) adj6 adeno-carcin*).mp,kw.	0	Adv
29	((fallopian tube? or tubal) adj6 malign*).mp,kw.	19	Adv
30	((fallopian tube? or tubal) adj6 metast*).mp,kw.	15	Adv
31	((fallopian tube? or tubal) adj6 oligo*).mp,kw.	6	Adv
32	((fallopian tube? or tubal) adj6 (recur* or re-cur*)).mp,kw.	238	Adv
33	((fallopian tube? or tubal) adj6 (reoccur* or re-occur*)).mp,kw.	0	Adv
34	((fallopian tube? or tubal) adj6 (relaps* or re-laps*)).mp,kw.	59	Adv
35	((fallopian tube? or tubal) adj6 progres*).mp,kw.	40	Adv
36	((fallopian tube? or tubal) adj6 onco*).mp,kw.	34	Adv
37	PFTC.mp,kw.	5	Adv
38	or/20-37	1353	Adv
39	Peritoneal Neoplasms/	354	Adv
40	Peritoneum/	307	Adv
41	(periton* adj6 neoplas*).mp,kw.	454	Adv
42	(periton* adj6 cancer*).mp,kw.	1772	Adv
43	(periton* adj6 tumo?r*).mp,kw.	391	Adv
44	(periton* adj6 carcin*).mp,kw.	865	Adv
45	(periton* adj6 adenocarcin*).mp,kw.	169	Adv
46	(periton* adj6 adeno-carcin*).mp,kw.	1	Adv
47	(periton* adj6 malign*).mp,kw.	199	Adv
48	(periton* adj6 metast*).mp,kw.	712	Adv
49	(periton* adj6 oligo*).mp,kw.	10	Adv
50	(periton* adj6 (recur* or re-cur*)).mp,kw.	614	Adv
51	(periton* adj6 (reoccur* or re-occur*)).mp,kw.	0	Adv
52	(periton* adj6 (relaps* or re-laps*)).mp,kw.	134	Adv

53	(periton* adj6 progres*).mp,kw.	239	Adv
54	(periton* adj6 onco*).mp,kw.	105	Adv
55	psammomacarcino*.mp,kw.	0	Adv
56	psammoma-carcino*.mp,kw.	0	Adv
57	or/39-56	3225	Adv
58	19 or 38 or 57	12368	Adv
59	"Neoplasms, Cystic, Mucinous, and Serous"/	11	Adv
60	exp Cystadenocarcinoma/	121	Adv
61	Cystadenocarcinoma, Serous/	97	Adv
62	exp Cystadenoma/	7	Adv
63	Cystadenoma, Serous/	2	Adv
64	"Neoplasms, Glandular and Epithelial"/	238	Adv
65	serous*.mp,kw.	1573	Adv
66	epithelial*.mp,kw.	9306	Adv
67	(low adj3 grade?).mp,kw.	4389	Adv
68	cystadeno*.mp,kw.	247	Adv
69	or/59-68	14824	Adv
70	58 and 69	2735	Adv
71	(low adj2 grade? adj5 serous*).mp,kw.	49	Adv
72	LGS*.mp,kw.	228	Adv
73	or/70-72	2944	Adv
74	Antibodies, Monoclonal, Humanized/	4397	Adv
75	Bevacizumab/	2107	Adv
76	bevacizumab*.mp,kw.	7237	Adv
77	abp 215.mp,kw.	20	Adv
78	abp215.mp,kw.	0	Adv
79	ask b1202.mp,kw.	0	Adv

80	askb1202.mp,kw.	1	Adv
81	abevmy*.mp,kw.	0	Adv
82	ainex*.mp,kw.	0	Adv
83	altuzan*.mp,kw.	1	Adv
84	alymsys*.mp,kw.	0	Adv
85	avastin*.mp,kw.	929	Adv
86	aybintio*.mp,kw.	0	Adv
87	bat 1706.mp,kw.	0	Adv
88	bat1706.mp,kw.	9	Adv
89	"bcd 021".mp,kw.	6	Adv
90	bcd021.mp,kw.	0	Adv
91	bevax*.mp,kw.	3	Adv
92	bevz 92.mp,kw.	0	Adv
93	bevz92.mp,kw.	7	Adv
94	bi 695502.mp,kw.	6	Adv
95	bi695502.mp,kw.	0	Adv
96	bryxta*.mp,kw.	0	Adv
97	chs 5217.mp,kw.	0	Adv
98	chs5217.mp,kw.	0	Adv
99	ct p16.mp,kw.	7	Adv
100	ctp16.mp,kw.	0	Adv
101	fkb 238.mp,kw.	0	Adv
102	fkb238.mp,kw.	5	Adv
103	hd 204.mp,kw.	1	Adv
104	hd204.mp,kw.	8	Adv
105	"hlx 04".mp,kw.	0	Adv
106	hlx04.mp,kw.	14	Adv

107	krabeva*.mp,kw.	0	Adv
108	kyomarc*.mp,kw.	0	Adv
109	lextemy*.mp,kw.	0	Adv
110	"mb 02".mp,kw.	1	Adv
111	mb02.mp,kw.	17	Adv
112	mil 60.mp,kw.	0	Adv
113	mil60.mp,kw.	5	Adv
114	mvasi*.mp,kw.	6	Adv
115	myl 14020.mp,kw.	0	Adv
116	myl14020.mp,kw.	0	Adv
117	myl 1402o.mp,kw.	10	Adv
118	myl1402o.mp,kw.	0	Adv
119	nsc 704865.mp,kw.	33	Adv
120	nsc704865.mp,kw.	1	Adv
121	onbevzi*.mp,kw.	0	Adv
122	ons 1045.mp,kw.	0	Adv
123	ons1045.mp,kw.	0	Adv
124	ons 5010.mp,kw.	2	Adv
125	ons5010.mp,kw.	0	Adv
126	oyavas*.mp,kw.	0	Adv
127	"pf 06439535".mp,kw.	13	Adv
128	pf06439535.mp,kw.	0	Adv
129	pf 6439535.mp,kw.	0	Adv
130	pf6439535.mp,kw.	0	Adv
131	ql 1101.mp,kw.	0	Adv
132	ql1101.mp,kw.	6	Adv
133	rg 435.mp,kw.	0	Adv

134	rg435.mp,kw.	0	Adv
135	rhuMAb-VEGF.mp,kw.	70	Adv
136	ro 4876646.mp,kw.	9	Adv
137	ro4876646.mp,kw.	79	Adv
138	sb 8.mp,kw.	6	Adv
139	sb8.mp,kw.	7	Adv
140	stc 103.mp,kw.	0	Adv
141	stc103.mp,kw.	0	Adv
142	zirabev*.mp,kw.	2	Adv
143	or/74-142	11098	Adv
144	73 and 143	317	Adv
145	(abstract or book or book article or book or book note or "book review" or book series article or book series article in press or book series chapter or book series conference paper or book series letter or "book series review" or book series short survey or chapter or conference abstract or conference proceeding or "conference review" or journal conference abstract or "journal conference review").pt.	206780	Adv
146	conferenc*.so.	41159	Adv
147	145 or 146	208693	Adv
148	144 not 147	220	Adv
149	limit 148 to yr="2021 -Current"	21	Adv

ClinicalTrials.gov February 15, 2022

LGS OR LGSOC OR serous OR epithelial OR low grade OR cystadenocarcinoma OR cystadenoma | ovarian OR fallopian OR peritoneal OR peritoneum | bevacizumab OR abevmy OR ainex OR altuzan OR alymsys OR avastin OR aybintio OR bevax OR bryxta OR krabeva OR kyomarc OR lextemy OR onbevzi OR oyavas OR zirabev | Studies with Female Participants | Adult, Older Adult

Results: 164 trials

International Standard Randomised Controlled Trial Number Registry (ISRCTN) February 15, 2022

Interventions: Bevacizumab
Condition Category: Cancer
Participant age range: Adult

Results: 22 trials

International Clinical Trials Registry Platform (ICTRP) February 15, 2022

Search History:
In the Condition: LGS OR LGSOC OR serous OR low grade OR ovarian OR fallopian OR peritoneal OR peritoneum
AND
In the Intervention: bevacizumab OR avastin

Results: 63 trials

Search results:

Recruitment status	Prospective Registration	Main ID	Public Title	Date of Registration	Results available
Authorised	No	EUCTR2020-004936-72-IT	A Study to Evaluate the Efficacy and Safety of Biomarker-Driven Therapies in Patients with Persistent or Recurrent Rare Epithelial Ovarian Tumors	2021-08-17	
Recruiting	Yes	NCT04931342	A Study Evaluating the Efficacy and Safety of Biomarker-Driven Therapies in Patients With Persistent or Recurrent Rare Epithelial Ovarian Tumors	2021-06-09	
Recruiting	Yes	NCT04938583	Oregovomab in Combination With Bevacizumab Plus Chemo in BRCA Wild Type Platinum Sensitive Recurrent Ovarian Cancer	2021-05-24	

Recruiting	Yes	KCT0006051	A single-arm phase II study of niraparib and bevacizumab maintenance therapy in platinum-sensitive recurrent ovarian cancer patients previously treated with a PARP inhibitor	2021-04-05
Recruiting	Yes	NCT04735861	Sintilimab Plus Bevacizumab in Recurrent/Persistent Ovarian Clear Cell Carcinoma	2021-01-29
Recruiting	Yes	NCT04734665	Niraparib and Bevacizumab Maintenance Therapy in Platinum-sensitive Recurrent Ovarian Cancer Patients Previously Treated With a PARP Inhibitor	2021-01-28
Authorised	Yes	EUCTR2020-000146-33-DE	Trial on NIraparib-TSR-042 (dostarlimab) vs physician's choice CHEmotherapy in recurrent, ovarian, fallopian tube or primary peritoneal cancer patients not candidate for platinum retreatment: NItCHE trial (MITO 33)	2020-11-18
Authorised	No	EUCTR2017-002860-42-IT	A randomized phase II trial of Carboplatin-Paclitaxel-Bevacizumab vs Carboplatin-Paclitaxel-Bevacizumab-Rucaparib vs Carboplatin-Paclitaxel-Rucaparib in patients with advanced (stage III B-C-IV) ovarian, primary peritoneal and Fallopian tube cancer preceded by a phase I dose escalation study on Rucaparib-Bevacizumab combination .	2020-11-04
Authorised	No	EUCTR2020-000146-33-IT	Trial on NIraparib-TSR-042 (dostarlimab) vs physician's choice CHEmotherapy in recurrent, ovarian, fallopian	2020-10-22

			tube or primary peritoneal cancer patients not candidate for platinum retreatment: NItCHE trial (MITO 33)	
Recruiting	Yes	NCT04679064	Trial on NIraparib-TSR-042 (Dostarlimab) vs Physician's Choice CHEmotherapy in Recurrent, Ovarian, Fallopian Tube or Primary Peritoneal Cancer Patients Not Candidate for Platinum Retreatment	2020-10-05
Recruiting	Yes	NCT04556071	Efficacy and Safety of Niraparib Combined With Bevacizumab in Platinum Refractory/Resistant Recurrent Ovarian Cancer	2020-09-15
Recruiting	Yes	KCT0005144	A single-arm phase II study of maintanance treatment of Olaparib, Pembrolizumab, and Bevacizumab in BRCA non-mutated patients with prior platinum-sensitive recurrent ovarian cancer	2020-06-17
Recruiting	Yes	NCT04361370	Olaparib Maintenance With Pembrolizumab & Bevacizumab in BRCA Non-mutated Patients With Platinum-sensitive Recurrent Ovarian Cancer	2020-04-19
Recruiting	Yes	ChiCTR2000030600	Study on the safety and effectiveness of cytoreductive surgery for colorectal cancer peritoneal metastasis patients who are sensitive to the first-line chemotherapy of FOLFOXIRI combined with bevacizumab	2020-03-08
Recruiting	Yes	NL8303	PIPAC and Systemic Chemotherapy for Irresectable Colorectal Peritoneal Metastases. (CRC-PIPAC-II)	2020-01-13

Recruiting	Yes	KCT0004506	Perfusion CT to Predict Progression-free Survival and Response Rate in Bevacizumab and Paclitaxel Treantment of Platinum-Resistant Persistent or Recurrent Epithelial Ovarian, Fallopian Tube, or Peritoneal Carcinoma	2019-12-06
Recruiting	Yes	NCT04175470	Bevacizumab and Tocotrienol in Recurrent Ovarian Cancer	2019-11-14
Authorised	Yes	EUCTR2019-000618-13-DK	Bevacizumab and tocotrienol in relapsed ovarian cancer	2019-05-10
Recruiting	Yes	JPRN-JapicCTI-184212	DUO-O	2018-11-21
Recruiting	Yes	NCT03737643	Durvalumab Treatment in Combination With Chemotherapy and Bevacizumab, Followed by Maintenance Durvalumab, Bevacizumab and Olaparib Treatment in Advanced Ovarian Cancer Patients	2018-10-15
Authorised	Yes	EUCTR2018-000413-20-FI	A study to determine whether the addition of TSR-042, followed by the use of niraparib with TSR-042, delays recurrence of ovarian, primary peritoneal or fallopian tube cancer	2018-08-28
Recruiting	Yes	NCT03587311	Bevacizumab and Anetumab Ravtansine or Paclitaxel in Treating Patients With Refractory Ovarian, Fallopian Tube, or Primary Peritoneal Cancer	2018-07-13
Not Recruiting	Yes	EUCTR2017-004456-30-CZ	STUDY TO EVALUATE THE EFFICACY AND SAFETY OF AVELUMAB IN COMBINATION WITH CHEMOTHERAPY FOLLOWED BY MAINTENANCE THERAPY OF	2018-06-29

			AVELUMAB IN COMBINATION WITH TALAZOPARIB IN PATIENTS WITH PREVIOUSLY UNTREATED ADVANCED OVARIAN CANCER	
Recruiting	Yes	NTR7177	Concomitant intraperitoneal and systemic chemotherapy in patients with extensive peritoneal carcinomatosis of colorectal origin	2018-04-26
Recruiting	Yes	NCT03462212	Carboplatin-Paclitaxel-Bevacizumab vs Carbo-Pacli-Beva-Rucaparib vs Carbo-Pacli-Ruca, Selected According to HRD Status, in Patients With Advanced Ovarian, Primary Peritoneal and Fallopian Tube Cancer, Preceded by a Phase I Dose Escalation Study on Ruca-Beva Combination	2018-02-14
Recruiting	Yes	NCT03363867	BEACON - ABC in Recurrent Platinum Resistant HGSOC	2017-11-28
Recruiting	Yes	NCT03353831	Atezolizumab With Bevacizumab and Chemotherapy vs Bevacizumab and Chemotherapy in Early Relapse Ovarian Cancer	2017-11-14
Authorised	Yes	EUCTR2015-005471-24-ES	A study of atezolizumab compared to placebo for subjects with late relapse ovarian cancer receiving a treatment with platinum-based chemotherapy and bevacizumab.	2017-10-10
Authorised	Yes	EUCTR2017-001982-26-GB	A study to compare the treatment of Vinblastine alone or in combination with Bevacizumab in children with progressive low grade glioma (LGG).	2017-09-21

Recruiting	Yes	NCT03095001	Intraperitoneal Chemotherapy Alone or in Combination With Bevacizumab for Ovarian Cancer With Peritoneal Adhesion	2017-03-16
Not recruiting	Yes	NCT03038100	A Study of Atezolizumab Versus Placebo in Combination With Paclitaxel, Carboplatin, and Bevacizumab in Participants With Newly-Diagnosed Stage III or Stage IV Ovarian, Fallopian Tube, or Primary Peritoneal Cancer	2017-01-30
Recruiting	Yes	NCT02884648	Bevacizumab in Ovarian Cancer Patients With Disease at Second-Look Surgery	2016-08-26
Recruiting	Yes	NCT02873962	A Phase II Study Of Nivolumab/ Bevacizumab/Rucaparib	2016-08-17
Recruiting	Yes	NCT02866903	Safety of Intraperitoneal (IP) OXAliplatin (OXA) in Association With Systemic FOLFIRI Bevacizumab Chemotherapy in Patients With Peritoneal Carcinosis	2016-08-04
Recruiting	Yes	NCT02839707	Pegylated Liposomal Doxorubicin Hydrochloride With Atezolizumab and/or Bevacizumab in Treating Patients With Recurrent Ovarian, Fallopian Tube, or Primary Peritoneal Cancer	2016-07-20
Authorised	Yes	EUCTR2015-005471-24-FR	A study of atezolizumab compared to placebo for subjects with late relapse ovarian cancer receiving a treatment with platinum-basedchemotherapy and bevacizumab.	2016-04-27
Recruiting	Yes	NCT02758951	Perioperative Systemic Therapy for Isolated	2016-04-26

				Resectable Colorectal Peritoneal Metastases	
Recruiting	Yes	NCT02840409	⊞	Vinblastine +/- Bevacizumab in Children With Unresectable or Progressive Low Grade Glioma (LGG)	2016-01-28
Not recruiting	Yes	NCT02659384	⊞	Anti-programmed Cell Death-1 Ligand 1 (aPDL-1) Antibody Atezolizumab, Bevacizumab and Acetylsalicylic Acid in Recurrent Platinum Resistant Ovarian Cancer	2016-01-14
Not Recruiting	Yes	EUCTR2015-002917-30-AT	⊞	Assessment of combination chemotherapy in colorectal cancer patients with peritoneal metastases by analysing obtained tumor tissue samples (CARCINOSIS).	2015-07-22
Recruiting	Yes	NCT02502266	⊞	Cediranib Maleate and Olaparib or Standard Chemotherapy in Treating Patients With Recurrent Platinum-Resistant or -Refractory Ovarian, Fallopian Tube, or Primary Peritoneal Cancer	2015-07-17
Recruiting	Yes	JPRN-UMIN000016619		A Feasibility Study of Gemcitabine and Bevacizumab in patients with Platinum-Resistant Recurrent Epithelial Ovarian, Primary Peritoneal, or Fallopian Tube Cancer	2015-03-01
Recruiting	Yes	NCT02364713		MV-NIS or Investigator's Choice Chemotherapy in Treating Patients With Ovarian, Fallopian, or Peritoneal Cancer	2015-02-10
Recruiting	Yes	NCT02312245		Avatar-Directed Chemotherapy in Treating Patients With Ovarian,	2014-12-04

				Primary Peritoneal, or Fallopian Tube Cancer	
Recruiting	Yes	NTR4632		Detectie van tumorweefsel met behulp van Bevacizumab-IRDye800CW in combinatie met een optisch beeldvormingssysteem bij patiënten die de HIPEC procedure ondergaan, een haalbaarheidsstudie.	2014-06-08
Recruiting	Yes	NCT01932125		An Interventional Study of Avastin (Bevacizumab) in Patients With Advanced/Metastatic Epithelial Ovarian Cancer, Fallopian Tube Cancer or Primary Peritoneal Cancer	2013-08-27
Not Recruiting	Yes	EUCTR2012-002841-39-DE	+	Evaluation of efficacy and safety of bevacizumab (Avastin®) combined to weekly paclitaxel followed by bevacizumab (Avastin®) alone versus weekly paclitaxel followed by observation in patients with relapsed ovarian sex-cord stromal tumours	2013-06-28
Recruiting	Yes	CTRI/2013/05/003627		A study to observe the safety of Bevacizumab when it is added to the standard chemotherapy (carboplatin and paclitaxel)when it is routinely prescribed to patients of advanced/metastatic epithelial ovarian,fallopian tube or primary peritoneal cancer	2013-05-10
Recruiting	No	NCT01838538		Clinical Study in Treatment of Malignant Ascites of Ovarian Cancer With Intraperitoneal Injection Bevacizumab Combined With Intraperitoneal	2013-04-19

			Hyperthermic Perfusion Chemotherapy		
Not recruiting	Yes	NCT01847274	A Maintenance Study With Niraparib Versus Placebo in Patients With Platinum Sensitive Ovarian Cancer	2013-04-11	Yes
Authorised	Yes	EUCTR2012-004362-17-IT	A multicenter phase III randomized study with second line chemotherapy plus or minus bevacizumab in patients with platinum sensitive epithelial ovarian cancer recurrence after a bevacizumab/chemotherapy first line	2012-12-21	
Not Recruiting	Yes	EUCTR2012-004125-24-DE	Evaluation of optimal treatment combination of bevacizumab in combination with gemcitabine/carboplatin or pegylated liposomal doxorubicin/carboplatin in patients with platin-sensitive recurrent ovarian cancer.	2012-11-29	
Authorised	Yes	EUCTR2012-003866-42-IT	multicenter italian study of the combination of bevacizumab and trabectedin with or without carboplatin in advanced ovarian cancer	2012-11-08	
Authorised	Yes	EUCTR2012-003043-29-IT	Clinical trial of first line treatment with carboplatin and paclitaxel plus bevacizumab for advanced ovarian cancer patients.	2012-09-14	
Recruiting	No	DRKS00003654	A Prospective Randomised Phase III Trial to Evaluate Optimal Treatment Duration of First-line Bevacizumab in Combination With Carboplatin and Paclitaxel in Patients With Primary Epithelial Ovarian, Fallopian Tube or Peritoneal Cancer	2012-04-30	

Authorised	Yes	EUCTR2010-022209-16-GB	ICON8 Trials Programme ICON8: Weekly chemotherapy in ovarian cancer, and ICON8B: Weekly chemotherapy and bevacizumab in advanced ovarian cancer	2010-12-30	
Not Recruiting	Yes	EUCTR2010-019525-34-SK	GLOBAL STUDY TO ASSESS THE ADDITION OF BEVACIZUMAB TO CARBOPLATIN AND PACLITAXEL AS FRONT-LINE TREATMENT OF EPITHELIAL OVARIAN CANCER, FALLOPIAN TUBE CARCINOMA OR PRIMARY PERITONEAL CARCINOMA - ROSiA	2010-08-23	
Authorised	Yes	EUCTR2009-017776-24-NL	A pilot study evaluating response to induction chemotherapy with oxaliplatin, capecitabine and bevacizumab in patients with extensive peritoneal carcinomatosis of colorectal origin.	2009-12-23	
Authorised	Yes	EUCTR2008-008336-85-FR	A single-arm Phase II clinical study of the combination of carboplatin and weekly paclitaxel plus bevacizumab as first-line treatment in patients with epithelial ovarian cancer - OCTAVIA	2009-03-25	
Not Recruiting	Yes	ISRCTN83438782	A GCIG Intergroup multicentre factorial trial of open label carboplatin and paclitaxel +/- bevacizumab compared with oxaliplatin and capecitabine +/- bevacizumab as first line chemotherapy in patients with mucinous Epithelial Ovarian Cancer	2008-07-30	Yes

Recruiting	No	NCT00418431	Intravitreal Bevacizumab for the Treatment of Central Serous Chorioretinopathy	2007-01-03
Authorised	Yes	EUCTR2005-003929-22-FR	ICON7 - A randomised, two-arm, multicentre Gynaecologic Cancer InterGroup trial of adding bevacizumab to standard chemotherapy (carboplatin and paclitaxel) in patients with epithelial ovarian cancer. - ICON7 GCIG Trial (International Collaborative Ovarian Neoplasm 7)	2006-11-27
Authorised	Yes	EUCTR2005-003209-10-FI	VEGF, VEGFR1 and VEGFR2 expression in serous epithelial ovarian carcinoma, and its association to the efficacy of bevacizumab combined with paclitaxel and carboplatin as first line treatment	2005-09-12
